# Distinct phenotypic states and spatial distribution of CD8^+^ T cell clonotypes in human brain metastases

**DOI:** 10.1016/j.xcrm.2022.100620

**Published:** 2022-04-27

**Authors:** Lisa J. Sudmeier, Kimberly B. Hoang, Edjah K. Nduom, Andreas Wieland, Stewart G. Neill, Matthew J. Schniederjan, Suresh S. Ramalingam, Jeffrey J. Olson, Rafi Ahmed, William H. Hudson

**Affiliations:** 1 Department of Radiation Oncology, Emory University School of Medicine, Atlanta, GA, USA; 2 Winship Cancer Institute, Emory University School of Medicine, Atlanta, GA, USA; 3 Department of Neurological Surgery, Emory University School of Medicine, Atlanta, GA, USA; 4 Emory Vaccine Center, Emory University School of Medicine, Atlanta, GA, USA; 5 Department of Microbiology and Immunology, Emory University School of Medicine, Atlanta, GA, USA; 6 Department of Pathology and Laboratory Medicine, Emory University School of Medicine, Atlanta, GA, USA; 7 Department of Hematology and Medical Oncology, Emory University School of Medicine, Atlanta, GA, USA

**Keywords:** brain metastases, CD8^+^ T cells, exhaustion, bystander, spatial transcriptomics, TCR-sequencing

## Abstract

Metastatic disease in the brain is difficult to control and predicts poor prognosis. Here, we analyze human brain metastases and demonstrate their robust infiltration by CD8^+^ T cell subsets with distinct antigen specificities, phenotypic states, and spatial localization within the tumor microenvironment. Brain metastases are densely infiltrated by T cells; the majority of infiltrating CD8^+^ T cells express PD-1. Single-cell RNA sequencing shows significant clonal overlap between proliferating and exhausted CD8^+^ T cells, but these subsets have minimal clonal overlap with circulating and other tumor-infiltrating CD8^+^ T cells, including bystander CD8^+^ T cells specific for microbial antigens. Using spatial transcriptomics and spatial T cell receptor (TCR) sequencing, we show these clonally unrelated, phenotypically distinct CD8^+^ T cell populations occupy discrete niches within the brain metastasis tumor microenvironment. Together, our work identifies signaling pathways within CD8^+^ T cells and in their surrounding environment that may be targeted for immunotherapy of brain metastases.

## Highlights

• Human brain metastases (BrMs) are well infiltrated by CD8^+^ T cells
• Exhausted CD8^+^ T cells in BrMs have little TCR overlap with other infiltrating cells
• Bystander CD8^+^ T cells inhabit BrMs and adopt a resident progenitor-like phenotype
• Spatial TCR sequencing shows CD8^+^ T cell localization is linked to phenotypic state

## Graphical abstract

**Figure:**
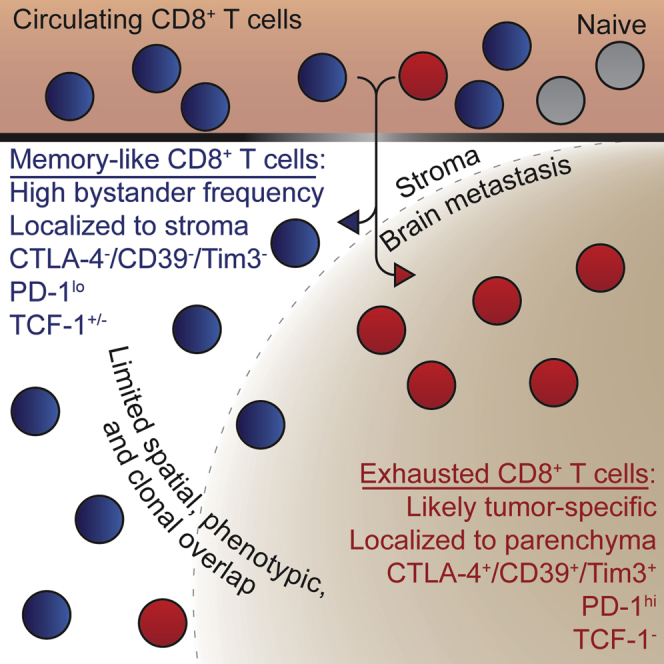

## Teaser

To develop improved immunotherapies for brain metastases, detailed characterization of their immune infiltrate is needed. Sudmeier et al. show that brain metastases are well infiltrated by CD8^+^ T cells, including bystander cells and exhausted, likely tumor-specific, cells. These cells exhibit distinct phenotypes and spatial localization within the brain metastasis tumor microenvironment.

## Introduction

The brain comprises a unique immune environment, classically thought to be immunosuppressive, that protects the central nervous system from excessive inflammation.^1^ Although there is loss of blood-brain-barrier (BBB) integrity within brain tumors,^2^^,^^3^ the consequent less-selective blood-tumor-barrier (BTB) and surrounding stroma of brain tissue may together affect inflammatory signaling and cell recruitment to the tumor microenvironment (TME) of brain metastases (BrMs). Tumor-specific CD8^+^ T cells, CD4^+^ T cells, and B cells have all been described in extracranial tumors,^4^, ^5^, ^6^, ^7^, ^8^ but the prevalence, phenotype, and antigen specificity of these cells in BrMs is unclear. For example, BrMs are infiltrated by CD8^+^ T cells^4^, ^5^, ^6^, ^7^, ^8^ that may not be clonally related to CD8^+^ T cells infiltrating patient-matched primary tumors,^9^ suggesting that BrMs may have unique determinants governing immune cell recruitment and response to immune checkpoint blockade (ICB) compared with the primary tumor. Here, we examine the lymphocytic infiltrate of BrMs, focusing on CD8^+^ T cells given their leading role in responsiveness to current immunotherapies.^10^

Exhausted CD8^+^ T cells, which are characterized by impaired proliferative and cytotoxic capacity,^11^ express inhibitory molecules such as PD-1, which further promote the exhausted phenotype.^12^ Inhibitory pathways like PD-1 are the targets of ICB agents, which block suppressive signaling in exhausted CD8^+^ T cells to rescue their proliferative and cytotoxic function.^10^ ICB agents have resulted in dramatic improvements in cancer disease control and patient survival; however, a significant proportion of cancer patients have refractory disease that either does not respond to ICB or progresses after an initial response.^13^^,^^14^ One strategy to improve ICB efficacy is to simultaneously block multiple inhibitory molecules expressed on exhausted CD8^+^ T cells.^15^ In order to identify potential therapeutic targets for this combination approach, a detailed phenotypic characterization of the target exhausted CD8^+^ T cells is required.

Exhausted CD8^+^ T cells are composed of diverse phenotypic subpopulations with distinct functions, inhibitory molecule expression, and tissue homing patterns. Exhausted progenitor PD-1^+^ CD8^+^ T cells are maintained by expression of the transcription factor TCF-1; they self-renew and produce daughter cells that undergo further differentiation.^16^, ^17^, ^18^ Transitory PD-1^+^ CD8^+^ T cells are the immediate progeny of these progenitor cells and are characterized by expression of effector molecules and loss of TCF-1 expression. Transitory cells are migratory; circulating antigen-specific CD8^+^ T cells in cancer and chronic infection are found in this state, which is marked by CX3CR1 expression.^19^, ^20^, ^21^, ^22^, ^23^, ^24^ Upon migration to non-lymphoid tissues, transitory cells further differentiate into a terminally differentiated population with increased expression of inhibitory molecules.^19^, ^20^, ^21^ These terminally differentiated CD8^+^ T cells are resident in non-lymphoid tissues, have poor effector function, and lack proliferative capacity.^19^^,^^21^

These CD8^+^ T cell populations each respond differently to PD-1 pathway blockade. ICB acts on effector CD8^+^ T cells at the site of antigen to improve their effector function by increasing their expression of molecules such as granzymes and perforins.^18^^,^^19^^,^^25^^,^^26^ The exhausted progenitor population is required for the proliferative burst observed after ICB, which produces significant expansion in the number of transitory effector cells.^16^, ^17^, ^18^ Thus, the progenitor population of exhausted CD8^+^ T cells has received significant attention in tumor immunology studies, many of which have quantified tumor-infiltrating TCF-1^+^ cells.^21^^,^^27^, ^28^, ^29^ However, the antigen specificity of tumor-infiltrating TCF-1^+^ CD8^+^ T cells is rarely determined.

Tumor-specific exhausted progenitor TCF-1^+^ CD8^+^ T cells have been found in human papillomavirus positive (HPV+) head and neck squamous cell carcinoma.^30^ Similar progenitor cells were also recently identified in melanoma and non-small cell lung cancers, where a majority of tumor-specific CD8^+^ T cells were in a terminally differentiated state.^31^^,^^32^ In murine models, antigen-specific exhausted progenitor CD8^+^ T cells are enriched in tumor-draining lymph nodes and are found exclusively in secondary lymphoid organs during chronic infection.^33^, ^34^, ^35^ However, it is unclear whether tumor-specific exhausted progenitor CD8^+^ T cells reside in tumors growing in the brain. In the absence of antigen-specificity information, CD8^+^ T cell function is often inferred from the phenotype. However, this approach is confounded by the expression of some molecular markers at multiple stages of CD8^+^ T cell differentiation. TCF-1, for example, is expressed by naive, memory, and exhausted progenitor CD8^+^ T cells. Cytomegalovirus (CMV)- and Epstein-Barr virus (EBV)-specific effector memory CD8^+^ T cells co-express TCF-1 and TOX,^36^ a transcription factor associated with T cell exhaustion.^37^, ^38^, ^39^ EBV- and influenza-specific CD8^+^ T cells have also been found in primary and metastatic brain tumors.^40^ Moreover, brain-resident memory cells have been reported to maintain antigen-independent PD-1 expression.^41^

Here, we describe the composition of BrM-infiltrating lymphocytes from a cohort of 31 patients and perform a detailed characterization of the CD8^+^ T cells and their surrounding TMEs in a subgroup of these patients. BrMs were well infiltrated by T cells, and the majority of CD8^+^ T cells expressed PD-1. Using single-cell RNA sequencing (scRNA-seq), we identified four transcriptional populations among PD-1^+^ CD8^+^ T cells infiltrating BrMs: dividing cells, terminally differentiated cells, and two clusters that shared some phenotypic features with exhausted progenitor cells. These first two subsets shared significant clonal overlap with each other but had minimal T cell receptor (TCR) overlap with the progenitor-like populations. We systematically identified bystander cells specific for microbial antigens among BrM-infiltrating CD8^+^ T cells; these were rare in the terminally differentiated and dividing populations and had a phenotype similar to that of exhausted progenitor cells. Bystanders were present among BrM-infiltrating PD-1^+^ CD8^+^ T cells and circulating PD-1^+^ CD8^+^ T cells at a similar frequency. To determine the location of specific CD8^+^ T cell clones within the tumor, we developed a method to obtain TCR sequences from spatial transcriptomics data^42^ and showed that CD8^+^ T cells from each population were spatially restricted to specific regions of the BrM TME; these regions contained distinct gene expression patterns and cytokine profiles. Together, our results show that BrMs are infiltrated by diverse populations of CD8^+^ T cells that adopt specific phenotypes and segregate to distinct niches within the TME based on their antigen specificity. These data may guide novel immunotherapeutic strategies for the treatment of BrMs.

## Results

### Human BrMs are well infiltrated by T cells

**Figure 1:**
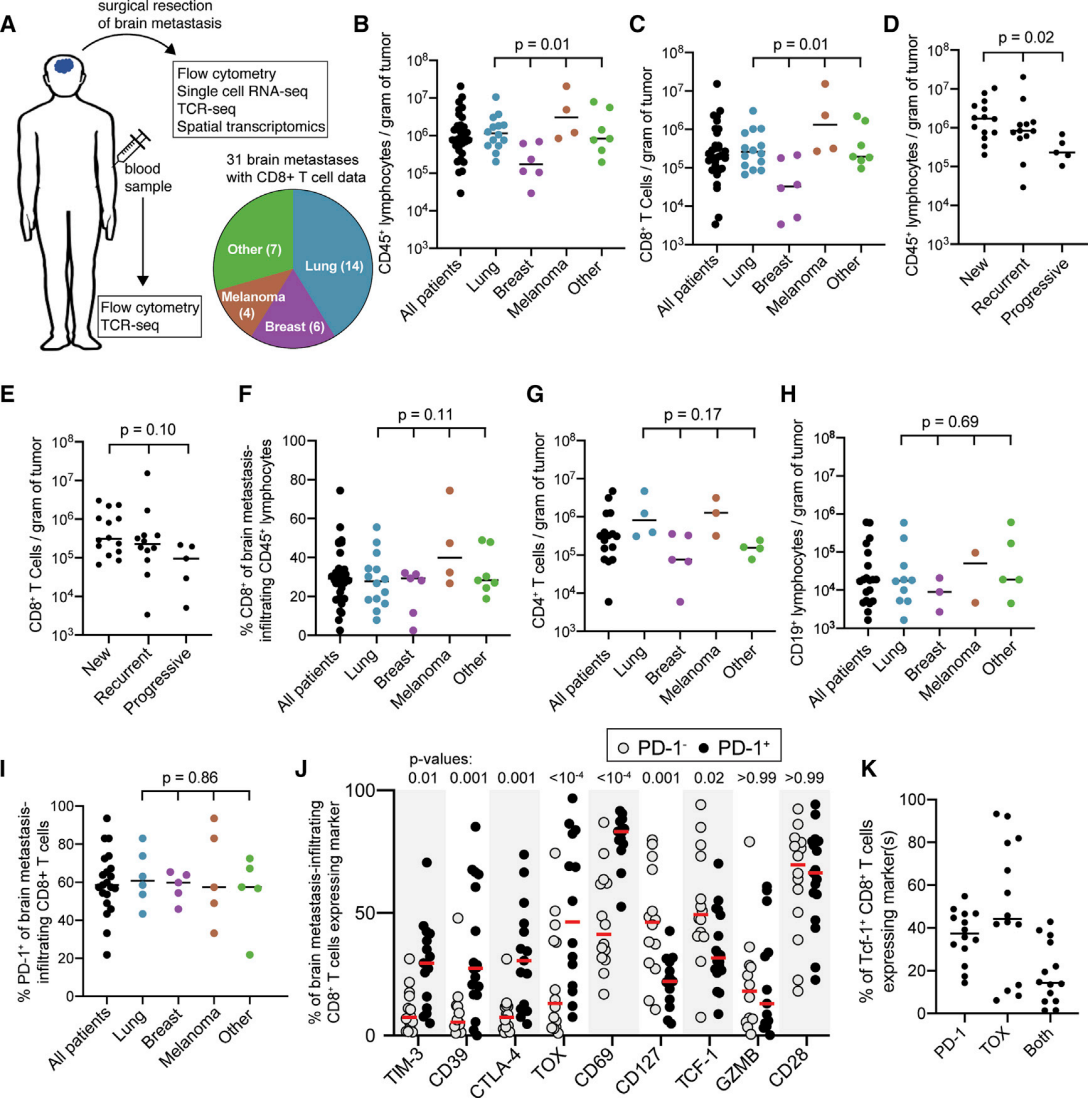
Human brain metastases are well infiltrated by PD-1^+^ CD8^+^ T cells (A) Experimental schema and distribution of samples among primary tumor histologies. (B and C) Density of CD45^+^ lymphocytes (B) and CD8^+^ T cells (C) for all 31 brain metastases grouped by primary tumor type. (D and E) Density of CD45^+^ lymphocytes (D) and CD8^+^ T cells (E) for all 31 brain metastases grouped by patient disease status at time of tumor resection. (F) Frequency of CD8^+^ cells among lymphocytes grouped by tumor type for all 31 patients. (G and H) Density of CD4^+^ T cells (G) and B cells (H) for 20 and 16 of the tumors, respectively. (I) Percentage of CD8^+^ T cells expressing PD-1 in 21 tumors. (J) Phenotype of PD-1^-^ versus PD-1^+^ CD8^+^ T cells within brain metastases (n = 12–14 for each marker). (K) Percentage of tumor-infiltrating TCF-1^+^ CD8^+^ T cells expressing PD-1, TOX, or both (n = 14). Bars on graphs indicate median. In (B)–(E) and (G), statistics show variance among primary tumor types with the Kruskal-Wallis test. In (F), (H), and (I), statistics show one-way ANOVA. In (J), statistics show a mixed-effects model analysis with Sidak’s multiple comparisons test. See also Figure S1.

Over 18 months, we collected fresh BrM specimens and matched blood samples from 31 patients at Emory University Hospital who underwent surgical resection of at least one BrM (Figure S1A). Samples were obtained fresh at time of surgery and included a mixture of primary tumor types, the most abundant of which was lung carcinoma (Figures 1A and S1A), consistent with it being the primary cancer most likely to metastasize to the brain.^43^^,^^44^ All patients were naive to immunotherapy. The immune infiltrate of all samples was quantified by flow cytometry. A subset of samples was used for high-parameter flow cytometry, scRNA-seq, TCR sequencing, and immunohistochemistry with spatially resolved transcriptomics (Figure 1A).

BrMs were variably infiltrated by immune cells, ranging from 2.9 × 10^4^ to 2 × 10^7^ CD45^+^ lymphocytes per gram of tumor (median 7.6 × 10^5^), with BrMs from breast cancer having the lowest infiltration density (Figures 1B and S1B). CD8^+^ T cell infiltration was also variable (range of 3.4 × 10^3^ to 1.5 × 10^7^, median of 2.0 × 10^5^ CD8^+^ T cells/gram of tumor), with breast-carcinoma histology again showing the lowest density of infiltration (Figures 1C and S1C). Most of the cohort presented with a new diagnosis of cancer or recurrence after previous definitive treatment; 5 patients had tumors resected after progressing in the brain while on systemic therapy (not immunotherapy). The density of CD45^+^ lymphocytes was lower in the tumors that progressed on treatment, and a similar trend was observed for CD8^+^ T cells (Figures 1D and 1E). Despite significant variability among samples, CD8^+^ T cells comprised a similar proportion of lymphocytes within BrMs, regardless of primary tumor type (Figure 1F). CD4^+^ T cell infiltration was similar to that of CD8^+^ T cells (range of 5.9 × 10^3^ to 4.7 × 10^6^, median of 3.1 × 10^5^ CD4^+^ T cells/gram of tumor), with lower infiltration of breast-carcinoma metastases (Figure 1G). Just under 10% of tumor-infiltrating CD4^+^ T cells expressed FOXP3 by flow cytometry across measured samples (Figure S1D). B cell infiltration was markedly lower than T cell infiltration (median of 1.8 × 10^4^ CD19^+^ cells/gram of tumor), a trend observed across all primary tumor types (Figure 1H).

### A subset of BrM-infiltrating CD8^+^ T cells have a progenitor phenotype

The response to PD-1 pathway blockade and other immunotherapies is primarily mediated by CD8^+^ T cells, and proliferation of these cells is associated with positive clinical outcomes after ICB.^10^^,^^45^ To interrogate the phenotype of circulating and tumor-infiltrating CD8^+^ T cells in patients with BrMs, we performed high-parameter flow cytometry on patient-matched tumor-infiltrating and circulating CD8^+^ T cells (Figure S2A). Despite some variation, a majority of BrM-infiltrating CD8^+^ T cells expressed PD-1; this was consistent across different tumor histologies (Figure 1I). We compared the expression of other co-inhibitory molecules, transcription factors, and the effector molecule granzyme B (GZMB) on PD-1^-^ and PD-1^+^ CD8^+^ T cells (Figure 1J). TOX and the co-inhibitory molecules TIM3, CD39, and CTLA-4 were significantly higher on PD-1^+^ cells compared with PD-1^-^ cells. Although CD69 was more highly expressed on PD-1^+^ cells, nearly half of PD-1^-^ cells also expressed CD69, indicating that a portion of these cells are also resident in the tumor (Figure 1J). While GZMB and the co-stimulatory molecule CD28 are expressed similarly between PD-1^+^ and PD-1^-^ CD8^+^ T cells, markers of progenitor function of CD8^+^ T cells such as CD127 and TCF-1 were higher in PD-1^-^ cells (Figure 1J).

Little is known about the abundance and phenotype of TCF-1^+^ CD8^+^ T cells in BrMs, and it is unclear whether tumor-specific exhausted progenitor cells reside in these tumors. CD127 and CD28 have been used as extracellular markers of TCF-1^+^ progenitor CD8^+^ T cells in chronic infections and cancer.^18^^,^^27^^,^^35^ In BrM-infiltrating CD8^+^ T cells, CD28 and CD127 were both more frequent on PD-1^+^ TCF-1^+^ compared with PD-1^+^ TCF-1^-^ cells (Figures S2B and S2C), but their expression did not completely recapitulate that of TCF-1. CD127 was mostly absent from PD-1^+^ TCF-1^-^ cells but only expressed on half of PD-1^+^ TCF-1^+^ cells (Figure S2B). CD28 was a more sensitive marker of TCF-1 expression and was found on over 75% of TCF-1^+^ cells but lacked specificity, with expression on over 50% of TCF-1^-^ cells (Figure S2B). The transcription factor TOX is a marker and regulator of CD8^+^ T cell exhaustion^37^, ^38^, ^39^ and is expressed on all tumor-specific CD8^+^ T cells in human cancers.^30^ In our cohort, 44% of BrM-infiltrating TCF-1^+^ CD8^+^ T cells expressed TOX and 37% were PD-1^+^; 14% of TCF-1^+^ CD8^+^ T cells co-expressed PD-1 and TOX (Figure 1K). Thus, despite the high frequency of TCF-1^+^ CD8^+^ T cells within BrMs, these cells are phenotypically diverse and TCF-1 expression alone may not be an adequate marker of tumor-specific CD8^+^ T cell progenitor function.

### Phenotypically distinct populations of CD8^+^ T cells infiltrate BrMs

**Figure 2:**
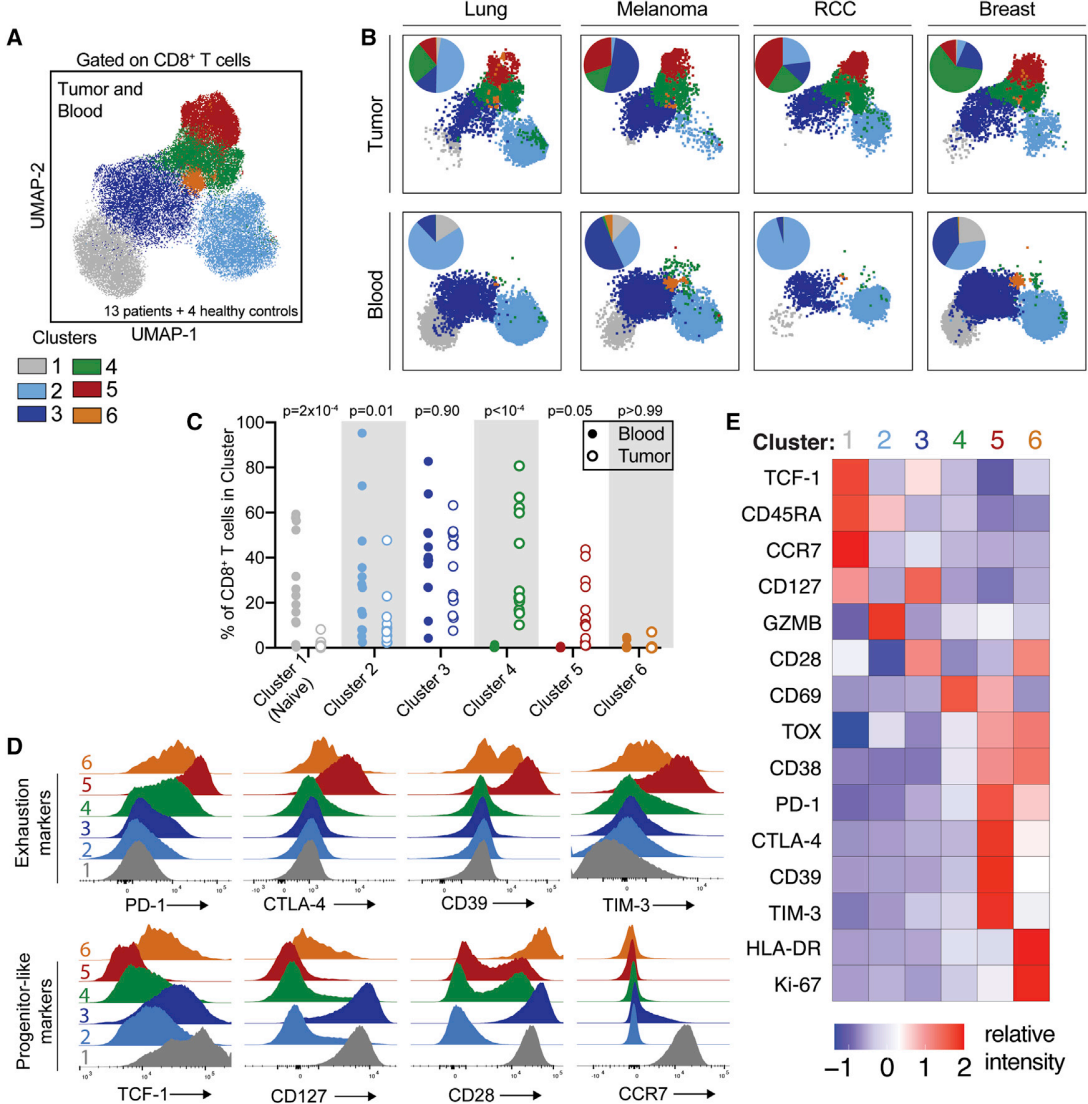
Spectral flow cytometry reveals that brain metastases are infiltrated by CD8^+^ T cells that are phenotypically distinct from circulating CD8^+^ T cells (A) Uniform manifold approximation and projections (UMAPs) of high-parameter flow-cytometry data gated on CD8^+^ T cells from 13 brain metastases, patient-matched blood, and blood from four healthy controls. Cells are colored by FlowSOM cluster. (B) UMAPs of CD8^+^ T cells from four individual patients (renal cell carcinoma [RCC]). Tumor-infiltrating and circulating CD8^+^ T cells are shown in the top and bottom rows, respectively. Inlaid pie charts show percentage of cells in each FlowSOM cluster. (C) Percentage of CD8^+^ T cells in each cluster is shown for each patient’s blood and tumor sample. (D) Expression of selected markers in each CD8^+^ T cell cluster. (E) Relative intensity of each indicated marker within each flow cluster. Statistics in (C) show differences between blood and tumor frequency by two-way ANOVA with Sidak’s multiple comparisons test. See also Figure S2.

FlowSOM clustering identified six populations of patient-matched tumor-infiltrating and circulating CD8^+^ T cells (Figure 2A). Cells from clusters 1 and 2 were preferentially found in blood (Figures 2B and 2C), indicating the presence of some selectivity for T cell infiltration across all BrMs. Cluster 3 was present in high frequencies in blood and tumor, clusters 4 and 5 were exclusively tumor infiltrating, and cluster 6 was a rare population in both blood and tumor (Figure 2C). Cluster 1 is composed of naive CD8^+^ T cells and is characterized by expression of CCR7 and CD45RA (Figures 2D and 2E); its exclusion from tumor samples is indicative of minimal blood contamination of BrM specimens. Cells in cluster 2 were predominately CD45RA^+^ and expressed high levels of GZMB (Figure 2E). Cluster 3 was present in the circulating and tumor compartments and was composed of heterogeneous PD-1^-^ and PD-1^dim^ cells with high levels of CD28, CD127, and TCF-1 (Figures 2C–2E). Cells in clusters 4 and 5 both expressed CD69, consistent with tissue residence and their predominance in the tumor (Figure 2E). Cells in both clusters 4 and 5 also expressed CD38 and were low for TCF-1 and CD127; expression of these latter two markers on intratumoral CD8^+^ T cells was primarily restricted to cluster 3, a cluster shared between the tumor and circulating compartments (Figures 2C–2E). Cluster 5 is an exhausted, terminally differentiated population of CD8^+^ T cells that expresses PD-1 as well as other co-inhibitory molecules including CTLA-4, CD39, and TIM-3 (Figures 2D and 2E). Most cells in cluster 5 also expressed TOX and were TCF-1^-^ (Figures 2E and S2D). Cells in cluster 6 are dividing (KI-67^+^) and express activation markers such as HLA-DR and CD38 as well as exhaustion markers such as PD-1 (Figure 2E). Cluster 6 cells express CD28 but low levels of TCF-1 and CD127 (Figures 2D and 2E). Together, these flow-cytometry results show that distinct and diverse CD8^+^ T cells populate BrMs; we next sought to characterize their gene expression profiles and antigen specificities.

### BrM-infiltrating CD8^+^ T cells comprise four metaclusters with distinct transcriptional phenotypes

**Figure 3:**
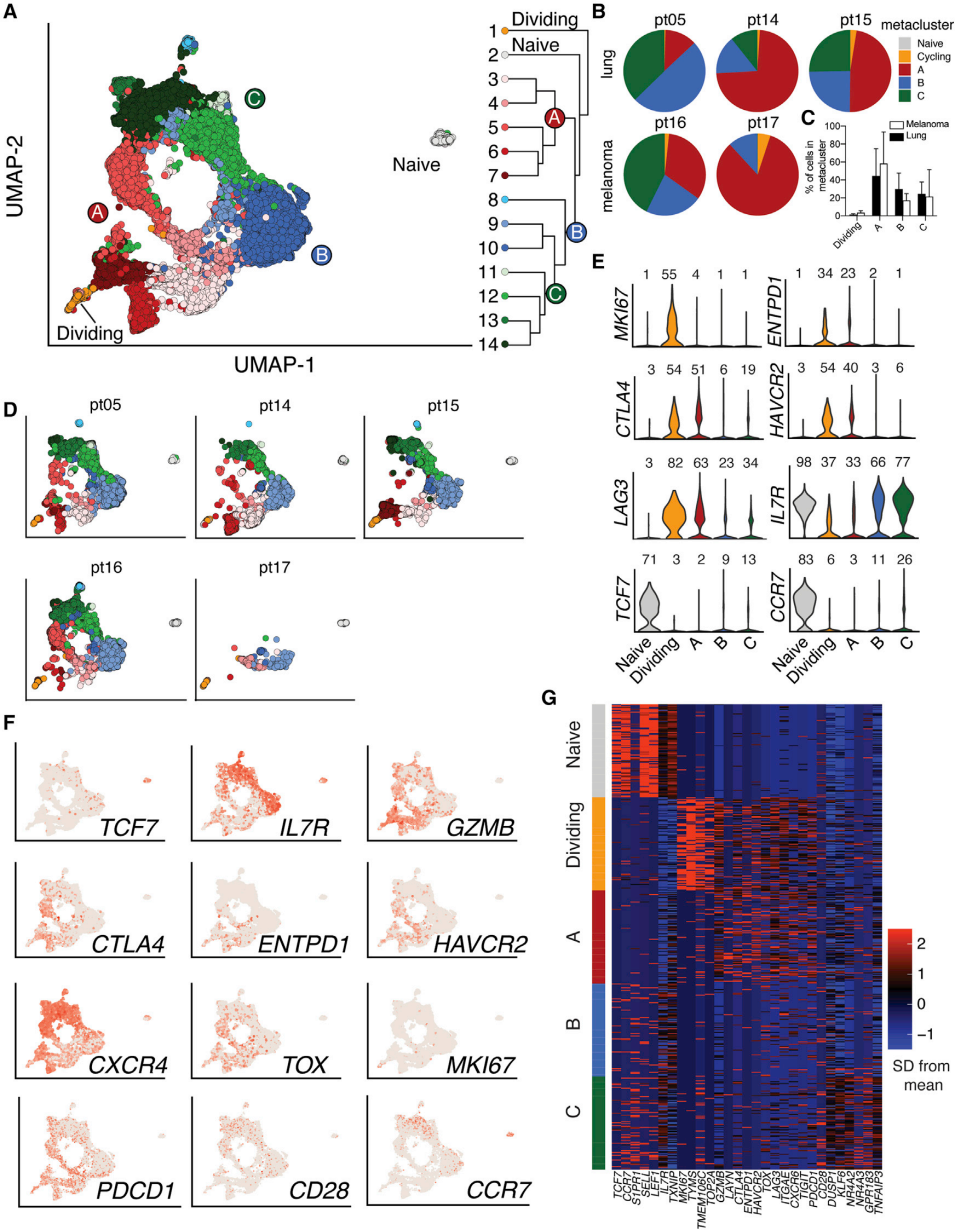
Transitory and terminally differentiated PD-1^+^ CD8^+^ T cells infiltrate human brain metastases (A) UMAPs of all 22,828 PD-1^+^ CD8^+^ T cells sorted from 5 brain metastases and naive cells sorted from patient-matched blood samples. Hierarchical clustering is shown at right. (B) Distribution of each patient’s PD-1^+^ CD8^+^ T cells among metaclusters. (C) Distribution of PD-1^+^ CD8^+^ T cells among metaclusters for each primary tumor type. (D) Phenotype of PD-1^+^ CD8^+^ T cells from brain metastasis and matched circulating naive cells for each patient. (E) Expression of selected genes in each metacluster. Numbers above each violin indicate the percentage of cells in each metacluster expressing the gene. (F) Expression of selected genes projected on UMAP. (G) Relative expression of selected genes in each metacluster. See also Figures S3 and S4.

CD8^+^ T cells specific for tumor-associated viral and neoantigen epitopes express PD-1.^30^^,^^46^, ^47^, ^48^ In our cohort, PD-1 expression within the tumor was highest on the terminally differentiated cluster 5 and dividing cells (FlowSOM cluster 6; Figures 2D and 2E). PD-1 was also expressed at lower levels in tumor-enriched cluster 4 and on some cells in cluster 3, which was shared between blood and tumor (Figures 2C–2E). To determine transcriptional profiles, interrogate differentiation pathways, and examine antigen specificity of these PD-1-expressing cells, we performed scRNA-seq with TCR sequencing on sorted PD-1^+^ CD8^+^ T cells isolated from three non-small cell lung carcinoma (NSCLC) BrMs and two melanoma BrMs immediately after surgical resection (Figure S3). These two histologies were chosen because they commonly metastasize to the brain.^43^ Naive CD8^+^ T cells were also sorted from patient-matched blood as a control. From 22,828 sequenced cells, we identified fourteen populations of PD-1^+^ CD8^+^ T cells, which were hierarchically clustered into 5 metaclusters with similar gene expression patterns: A, B, C, dividing (D), and naive (Figure 3A). Four of the five patients had cells in each of clusters A–D, with the exception being patient 17, who did not have cells in metacluster C (Figure 3B). There was no difference in the percentage of cells in each metacluster between BrMs from lung cancer versus melanoma, suggesting that these two tissues of origin did not strongly influence the phenotype of BrM-infiltrating PD-1^+^ CD8^+^ T cells here (Figures 3C and 3D), consistent with our flow-cytometry results (Figure S2E).

Tumor-infiltrating PD-1^+^ CD8^+^ T cells in the A and D metaclusters had a terminally differentiated phenotype similar to that of FlowSOM cluster 5 that was characterized by high expression of genes encoding co-inhibitory molecules such as *CTLA4*, *ENTPD1* (CD39), *HAVCR2* (TIM-3), and *LAG3*, consistent with this population containing tumor-reactive cells (Figures 3E, 3F, and S4A). These cells expressed a unique repertoire of genes encoding cell-surface proteins that could be assessed for co-inhibitory or co-stimulatory potential (Figure S4D). Cells in the D metacluster were additionally defined by high expression of cell-cycle genes such as *MKI67* (KI-67) and *TOP2A* (Figures 3E–3G). Metaclusters B and C expressed the lowest levels of co-inhibitory markers (Figures 3E, 3F, and S4). They were distinguished from each other by higher expression of tissue-residence genes, such as CD69, in metacluster C (Figure S4A). Together, metaclusters B and C contained PD-1^+^ CD8^+^ T cells with higher expression of the progenitor markers *TCF7* (TCF-1) and *IL7R* (CD127) compared with terminally differentiated and dividing cells (Figures 3E–G). Gene set enrichment analysis (GSEA) revealed that the transitory transcriptional signature characterized in the murine lymphocytic choriomeningitis virus (LCMV) model of CD8^+^ T cell exhaustion^19^ was enriched in the D metacluster, suggesting that these cells may be undergoing differentiation from stem-like to terminally differentiated PD-1^+^ CD8^+^ T cells (Figure S4B). Similar analysis showed that the LCMV terminally differentiated signature was enriched in metacluster A (Figure S4B). Thus, we hypothesized that metaclusters B and C may contain exhausted progenitor cells, while metaclusters A and D contained their terminally differentiated progeny.

### Terminally differentiated CD8^+^ T cells have minimal clonal overlap with circulating or progenitor-like tumor-infiltrating CD8^+^ T cells

**Figure 4:**
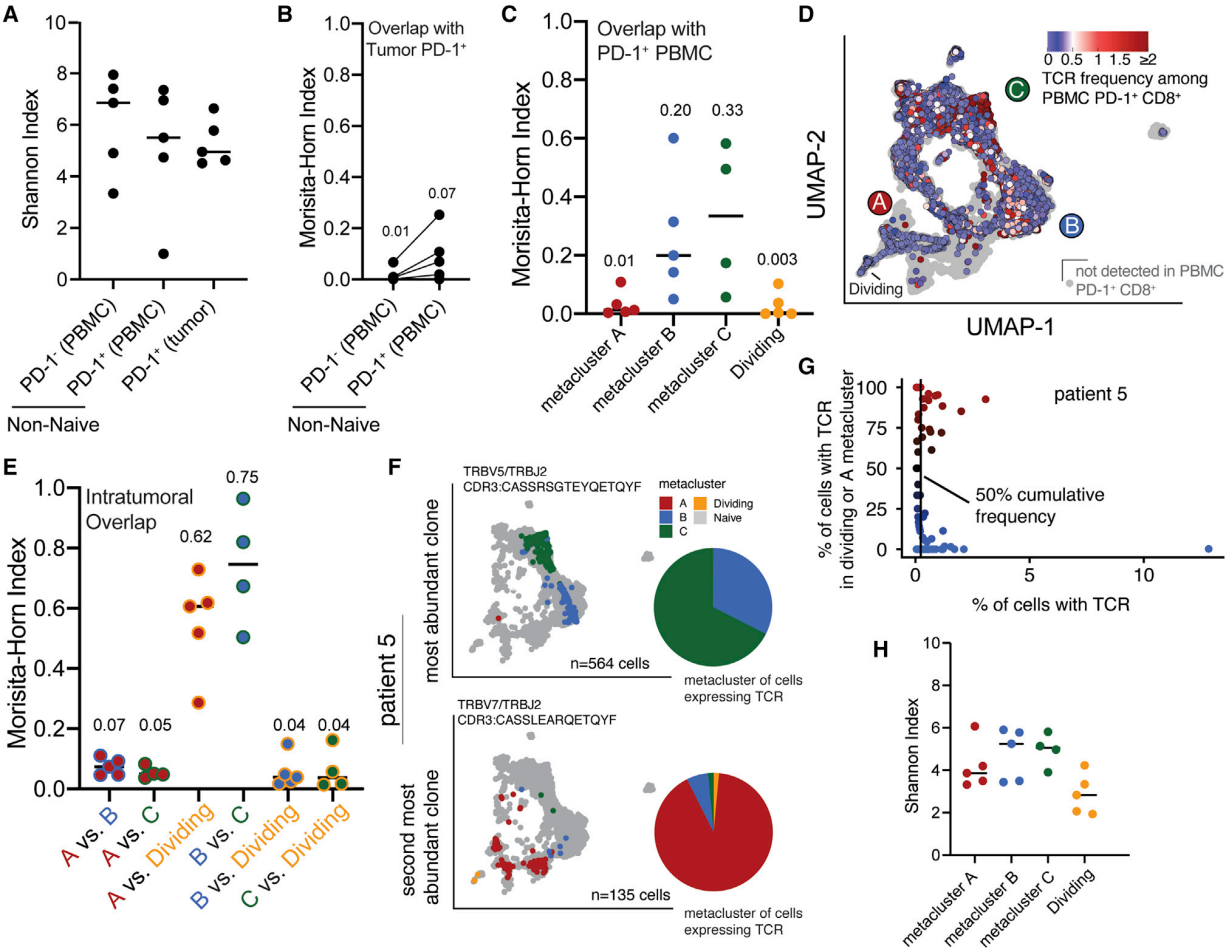
Dividing and terminally differentiated CD8^+^ T cells are clonally related to each other but not to other PD-1^+^ brain-metastasis-infiltrating or circulating CD8^+^ T cells (A) TCR diversity of circulating and brain-metastasis-infiltrating CD8^+^ T cells from the same 5 patients shown in Figure 3. (B) Quantification of TCR overlap between tumor-infiltrating PD-1^+^ CD8^+^ T cells with circulating PD-1^-^ and PD-1^+^ cells from individual patients. (C) Quantification of TCR overlap between circulating PD-1^+^ CD8^+^ T cells and tumor-infiltrating PD-1^+^ CD8^+^ T cells within each metacluster. (D) UMAPs colored by frequency of each cell’s TCR among circulating PD-1^+^ CD8^+^ T cells. (E) Quantification of TCR overlap between PD-1^+^ CD8^+^ T cells within each brain-metastasis-infiltrating metacluster. (F) UMAP of cells from the most abundant (top) and second most abundant (bottom) TCR clonotype in patient 5, colored according to metacluster. Gray dots represent all other cells from the patient. Pie charts show distribution among metaclusters of cells expressing the TCR. (G) Distribution in phenotype of all clones detected among brain-metastasis-infiltrating PD-1^+^ CD8^+^ T cells from patient 5. Vertical line indicates 50% cumulative frequency of TCR clones. (F and G) Four other patients are shown in Figure S6. (H) TCR diversity of intratumoral metaclusters. In (A), (C), (E), and (G), lines indicate the median. See also Figure S5.

To determine the clonal relationship between circulating and BrM-infiltrating CD8^+^ T cells, we performed TCR sequencing on non-naïve PD-1^-^ and PD-1^+^ CD8^+^ T cells sorted from patient-matched peripheral blood (Figure S3). Compared with circulating PD-1^-^ CD8^+^ T cells, circulating PD-1^+^ CD8^+^ T cells had lower TCR diversity and more overlap with tumor-infiltrating cells (Figures 4A and 4B). However, the overall overlap between circulating and tumor-infiltrating CD8^+^ T cells was minimal, suggesting that circulating CD8^+^ T cells expressing tumor-enriched TCRs are rare (Figure 4B).^24^^,^^49^ Circulating tumor-specific CD8^+^ T cells appear to be even more infrequent, as TCRs from terminally differentiated cells were rarely found in blood (Figures 4C and 4D). Tumor-infiltrating cells that did express blood-enriched TCRs were predominantly located in metaclusters B and C (Figures 4C and 4D). This is consistent with our flow-cytometry data, where TCF-1 and CD127 expression in the tumor was restricted to FlowSOM cluster 3, a population of cells shared between blood and tumor (Figures 2C and 2D).

To interrogate the differentiation pathways available to BrM-infiltrating PD-1^+^ CD8^+^ T cells, we analyzed TCR overlap among scRNA-seq metaclusters. In particular, we were interested in clonal overlap between metaclusters B/C and A/D, which would indicate *in situ* differentiation of progenitor-like CD8^+^ T cells to terminally differentiated cells within the BrM TME. Terminally differentiated and dividing cells (metaclusters A and D, respectively) had substantial TCR overlap with each other (Figure 4E). However, these dividing and exhausted cells exhibited minimal TCR overlap with metaclusters B and C, which contained cells with a less- or non-exhausted phenotype (Figures 4E and S5). Most CD8^+^ T cell clones—and particularly the most abundant clones—within each patient’s tumor were mostly restricted to either an exhausted (metaclusters A/D) or progenitor-like (metaclusters B/C) phenotype (Figures 4F, 4G, and S5), indicating that these populations have largely unshared antigen specificity. TCR diversity was also lower among terminally differentiated cells (metaclusters A/D) compared with cells in metaclusters B/C (Figure 4H). These data suggest that there are two distinct pools of exhausted (metacluster A/D) and memory-like (metaclusters B/C) PD-1^+^ CD8^+^ T cells between which differentiation is relatively restricted within the BrM TME. Our data do not preclude the presence of tumor-specific exhausted progenitor CD8^+^ T cells in metaclusters B/C but suggest that they are rare and that the majority of terminally differentiated CD8^+^ T cells (metaclusters A/D) appear to arise from exhausted progenitors outside of the tumor. We thus hypothesized that cells in metaclusters B and C may largely instead be bystander CD8^+^ T cells specific for non-tumor antigens that have become resident within the tumor following migration from the circulation.

### BrM-infiltrating bystander CD8^+^ T cells have phenotypic similarities to exhausted progenitor CD8^+^ T cells

**Figure 5:**
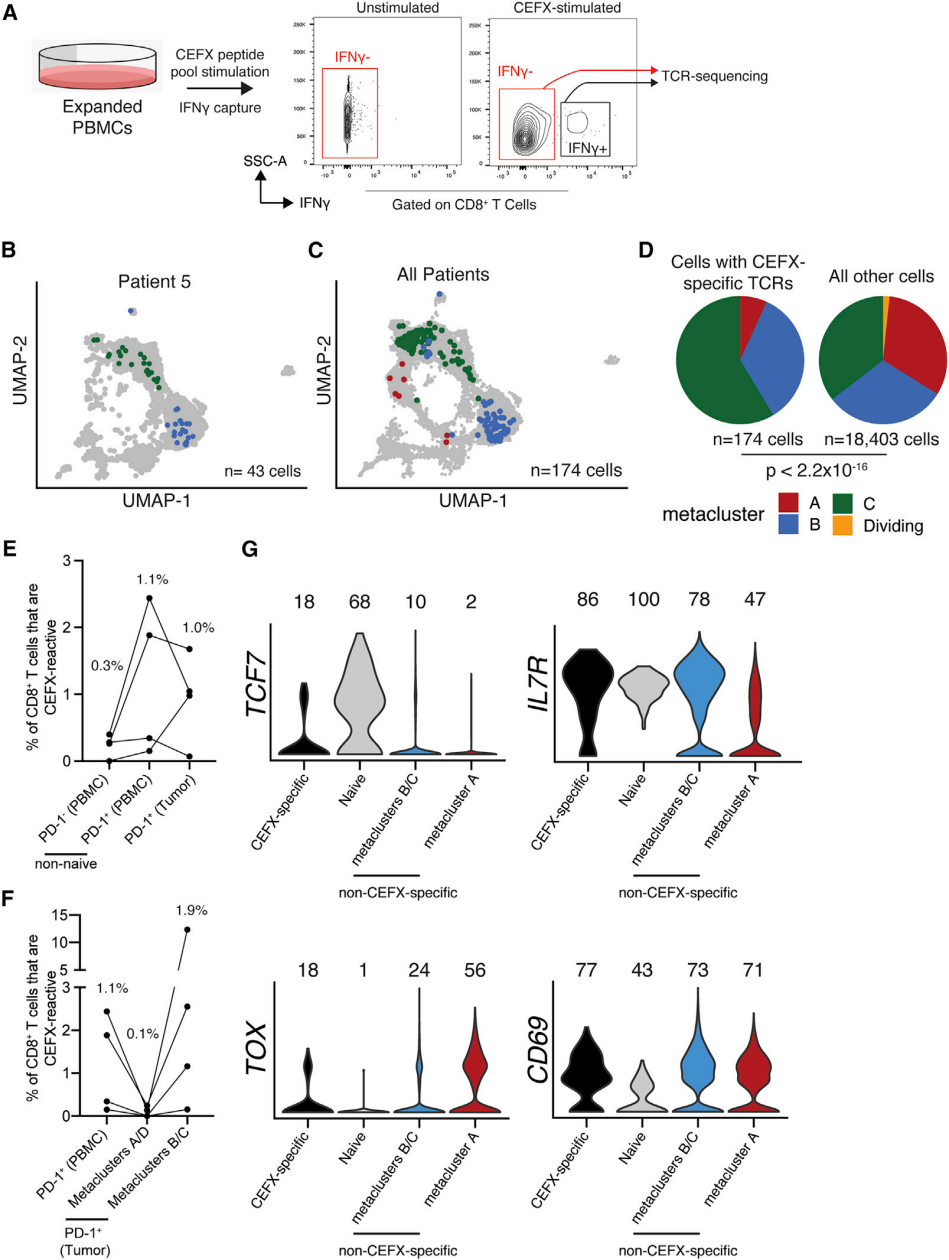
Microbe-specific CD8^+^ T cells are present in human brain metastases and are enriched in metaclusters B/C (A) We performed IFNγ capture on expanded, CEFX-stimulated PBMCs and subsequently sorted IFNγ^-^ and IFNγ^+^ CD8^+^ T cells for TCR sequencing to identify CEFX-specific TCRs from 4 of the 5 patients from whom we had scRNAseq data. (B and C) Brain-metastasis-infiltrating PD-1^+^ CD8^+^ T cells with CEFX-specific TCRs colored by phenotype on the UMAP for patient 5 (B) and all patients (C). (D) scRNA-seq phenotype of CEFX-specific (left) and all other (right) brain-metastasis-infiltrating PD-1^+^ CD8^+^ T cells from all four patients. (E) Frequency of CEFX-specific cells among circulating and tumor-infiltrating CD8^+^ T cells. (F) Frequency of CEFX-specific cells among circulating PD-1^+^ CD8^+^ T cells and tumor-infiltrating PD-1^+^ CD8^+^ T cell subsets. (G) Expression of selected genes by CEFX-specific brain-metastasis-infiltrating PD-1^+^ CD8^+^ T cells and all remaining cells by metacluster. In (E) and (F), medians are shown above each column. In (G), numbers indicate percentage of cells with measurable expression of the indicated marker. p value in (D) was calculated by Fisher’s exact test. See also Figures S6 and S7.

Previous studies have identified tumor-infiltrating bystander CD8^+^ T cells,^40^^,^^50^ but little is known about bystander infiltration of BrMs. We queried the VDJdb, a database of TCRs with known specificity,^51^ for matches with TCRs from our scRNA-seq data and identified one CMV-specific TCR in each of two patients (Figure S6). Both CMV-specific TCRs were found exclusively in cells within metaclusters B/C. To experimentally interrogate the abundance and phenotype of BrM-infiltrating bystander CD8^+^ T cells, we *ex vivo* expanded peripheral blood mononuclear cells (PBMCs) of four patients from whom scRNA-seq data were available. We stimulated these cells with a microbial peptide pool (CEFX), isolated interferon gamma (IFNγ)^+^ and IFNγ^-^ CD8^+^ T cells by fluorescence-activated cell sorting (FACS), and performed TCR sequencing on each subset to identify TCRs that responded to CEFX stimulation with cytokine secretion and were thus microbe specific (Figure 5A and S7A).

Comparison of TCR sequences from this assay and those identified from the scRNA-seq data above revealed that CEFX-specific cells ranged from 0.07% to 1.70% of BrM-infiltrating PD-1^+^ CD8^+^ T cells. Phenotypically, CEFX-specific BrM-infiltrating PD-1^+^ CD8^+^ T cells were found within metaclusters B/C in all four patients, where they were significantly enriched (Figures 5B–5D and S7B). A median of 1.9% of metacluster B/C cells were CEFX specific compared with 0.07% of metacluster A/D cells and 1.1% of total circulating PD-1^+^ CD8^+^ T cells (Figures 5E and 5F). Of note, 12.3% of metacluster B/C cells from patient 17 were CEFX specific. Importantly, some of these experimentally validated bystander cells had a transcriptional phenotype similar to tumor-specific progenitor PD-1^+^ CD8^+^ T cells characterized in other studies,^35^ marked by expression of *IL7R* (CD127), *TOX*, and *TCF7* (TCF-1) (Figures 5G and S7C). Of PD-1^+^ CD8^+^ T cells within the tumor, *IL7R* and *TCF7* expression was highest on CEFX-specific cells (Figure 5G).

Given the small number of known microbial T cell epitopes tested by this approach and the similar frequencies of bystander, non-tumor-specific cells between circulating and tumor-infiltrating PD-1^+^ T cells (Figure 5E), the frequency of total bystander cells within human BrMs is likely very high. Additionally, given the enrichment of CEFX-specific cells in metaclusters B/C compared with both tumor-infiltrating terminally differentiated cells and circulating cells (Figure 5F), it is probable that many cells in metaclusters B/C are specific for non-tumor antigens and do not give rise to cells with a terminally differentiated phenotype within the tumor. While these data do not preclude the presence of a small tumor-specific exhausted progenitor population within BrMs, a large fraction of tumor-infiltrating TCF-1^+^ CD8^+^ T cells appear to be bystander cells specific for non-tumor antigen.

### CD8^+^ T cell phenotype is linked with spatial distribution within the tumor

**Figure 6:**
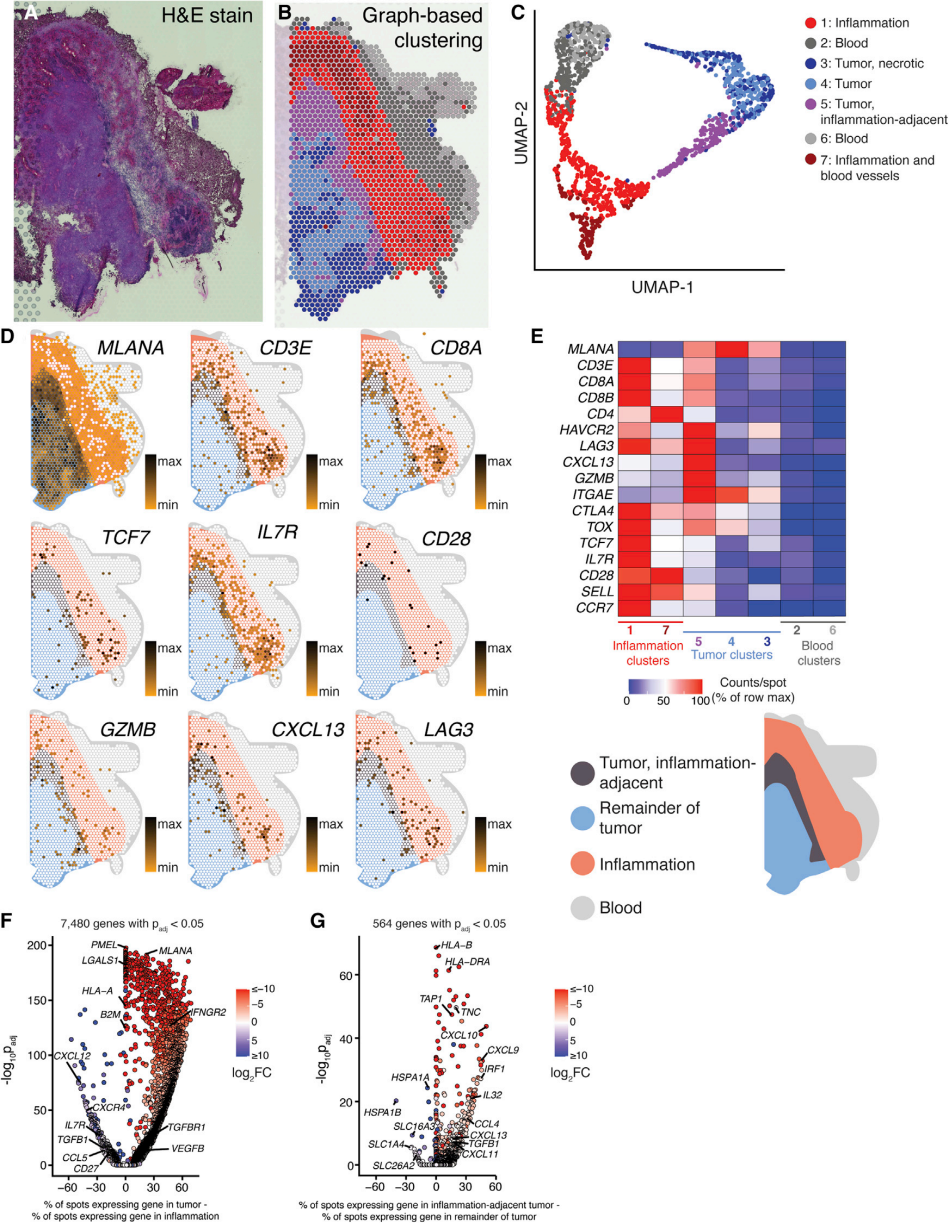
Genes associated with the terminally differentiated CD8^+^ T cell phenotype are preferentially expressed within the tumor parenchyma of a melanoma brain metastasis (A) Hematoxylin-and-eosin-stained section of a melanoma brain metastasis (patient 16). (B) Spatial location of capture spots, colored by transcriptional cluster. (C) UMAP and clustering of capture areas by transcriptional phenotype. (D) Expression of selected genes within the tissue section. White indicates no detection of the indicated gene in a given capture area. A spatial legend is given at bottom right. (E) Normalized expression density of selected genes in each tissue cluster. (F) Gene-expression differences between tumor (clusters 3, 4, and 5) and peritumoral inflammation (clusters 1 and 7). (G) Differential gene expression between inflammation-adjacent tumor (cluster 5) and the remainder of tumor (clusters 3 and 4). See also Figures S8–S14.

Given their divergent phenotypes and antigen specificity, we hypothesized that each subset of BrM-infiltrating CD8^+^ T cells may be located within distinct regions of the TME and thus receiving different signals from surrounding tissue. To test this, we performed spatial transcriptomics, a method to measure gene expression *in situ*, on six BrM tissue sections: one melanoma (Figure 6), one renal cell carcinoma (Figure S8), one breast carcinoma (Figure S9), and three lung carcinomas (Figures S10–S12). Capture spots were clustered based on gene expression, and each cluster was annotated based on appearance in H&E staining.

Tumor parenchyma—referring to regions of tumor cells—was readily differentiated by their gene expression profiles (Figures 6A, 6B, and S8–S12). For sections where tumor-normal boundaries were clearly demarcated, the number of differentially expressed genes between tumor parenchyma and surrounding stroma ranged from 3,914 to 7,480 (Figures 6F, S11C, and S12E). In the two samples where tumor parenchyma and stroma were intermixed, the numbers of differentially expressed genes between the two regions were 637 and 2,327 (Figures S8E and S9E). One of the six tissue sections was composed entirely of tumor parenchyma, precluding this analysis (Figures S10A–S10C).

The interplay between TME heterogeneity and immune infiltration was apparent. In the renal cell carcinoma case, “small nests” of tumors showed a higher density of *CD8A*, *CD8B*, and *CD4* expression than “large nests,” and these transcript levels were even higher in regions of desmoplasia surrounding vessels (Figures S8C and S8D). Additionally, in the two lung-carcinoma samples where tumor parenchyma and brain tissue were visible, *CD3E* transcript levels were highest at the tumor interface with brain (Figures S11D, S12C, and S12D). In the example of a melanoma BrM tissue section where tumor parenchyma was surrounded by inflammatory stroma (patient 16), densities of *CD3E*, *CD4*, *CD8A*, and *CD8B* transcripts were highest in the peritumoral inflammation and in the directly adjacent tumor parenchyma (Figures 6D and 6E). Together, these results are consistent with previous observations that immune cells are enriched in the peripheral region of BrM compared with the tumor core.^7^ In this melanoma sample, expression of genes associated with the terminally differentiated phenotype of CD8^+^ T cells, such as *HAVCR2* (TIM-3), *LAG3*, *CXCL13*, and *GZMB*, was highest in the tumor parenchyma adjacent to inflammation. Conversely, expression of the progenitor markers *TCF7* (TCF-1) and *IL7R* (CD127) was highest in the inflammatory stroma, suggesting that the immune cell phenotype determines its location within the diverse TME (Figures 6D and 6E).^7^ Together, these data suggest a linkage between the CD8^+^ T cell phenotype and the location within the BrM TME, with exhausted CD8^+^ T cells enriched in the tumor itself.

### Terminally differentiated CD8^+^ T cell clones are preferentially located in the tumor parenchyma

**Figure 7:**
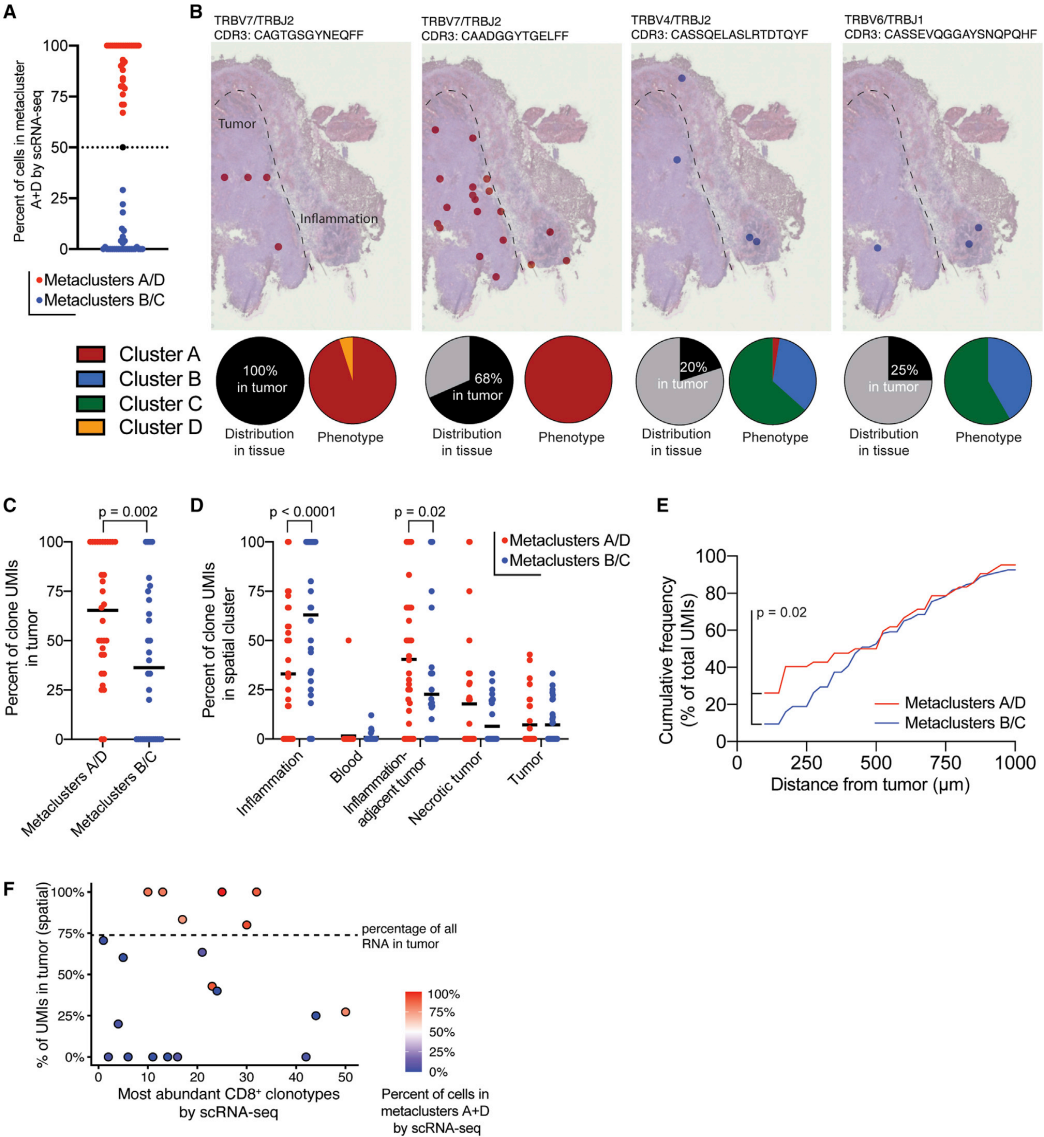
CD8^+^ T cell phenotype dictates location in the tumor microenvironment (A) scRNA-seq phenotype of brain-metastasis-infiltrating PD-1^+^ CD8^+^ T cells expressing TCRs identified by spatial transcriptomics in patient 16. Each dot represents one TCR clone identified in both scRNA-seq and spatial-transcriptomics data. For subsequent analysis, TCR clones were classified as metacluster A/D clones (red) or metacluster B/C clones (blue) based on the scRNA-seq phenotype of cells expressing the clone. (B) Spatial location of selected TCR clones within tissue from patient 16. Each dot represents a capture area in which at least one unique molecular identifier (UMI) for the indicated clone was found. Pie charts indicate the percentage of UMIs found in the tumor parenchyma (left) and the percentage of cells expressing the indicated TCR in each scRNA-seq metacluster (right). (C) Spatial distribution of UMIs from metacluster A/D and B/C clones in the tissue section shown in (B). Each dot is a single TCR clone. (D) Distribution of TCR UMIs in tissue clusters from patient 16 identified by spatial transcriptomics (Figure 6B). (E) Cumulative frequency of UMIs outside the tumor parenchyma as a function of distance from the tumor border. (F) Tumor localization of most frequent clones. Lower numbers indicate more expanded clones. TCRs not identified within the tissue section are not shown. Dashed line indicates the percentage of all gene expression UMIs found in tumor regions. The p value in (C) was calculated by Mann-Whitney test. p values in (D) were calculated by two-way ANOVA with Sidak’s multiple comparisons test. The p value in (E) was calculated by the Kolmogorov-Smirnov test. See also Figure S10.

However, genes that define these CD8^+^ T cell phenotypes may also be expressed by other cells within the TME, confounding this interpretation of our spatial-transcriptomics data. Because we have shown that TCR clones in BrMs are phenotypically restricted—that is, cells expressing a single TCR are predominately within scRNA-seq metaclusters A/D or B/C but not both (Figures 4E, 4F, and 7A)—localization of TCRs within the tumor would allow for visualization of specific CD8^+^ T cell phenotypes within the tumor. To determine whether there is spatial restriction of CD8^+^ T cell clones in the BrM TME, we developed and validated a method to amplify TCR transcripts from spatial-transcriptomics gene-expression libraries.^42^ By linking TCR clones found with this method and in our scRNA-seq data, we could determine the precise location of CD8^+^ T cells with specific transcriptional phenotypes within the TME (Figure 7B).

Of the six tissues on which we performed spatial transcriptomics, scRNA-seq data from fresh tissue were available for two: patients 15 and 16, with lung carcinoma and melanoma samples, respectively. In the melanoma sample (patient 16), we observed that CD8^+^ T cell clones with a metacluster A/D phenotype were predominantly located in the tumor parenchyma, while CD8^+^ T cell clones with a metacluster B/C phenotype were predominantly found in the peritumoral inflammation (Figures 7B–D). Not only were metacluster A/D TCRs enriched in the tumor parenchyma, but TCR clones with this phenotype found outside of the tumor were also preferentially located closer to the tumor boundary compared with those with a metacluster B/C phenotype (Figure 7E). When only the most expanded clones were considered, this difference in localization was more striking: 79% of metacluster A/D clones were found in the tumor versus 23% of metacluster B/C clones (Figure 7F). These findings were confirmed in the lung carcinoma BrM (patient 15), where the entire tissue section was tumor parenchyma and was inhabited only by TCRs expressed by metacluster A/D cells (Figures S10E–S10J).

Given this preference of specific CD8^+^ T cell subsets for particular locations within the TME, we sought to determine whether they receive distinct signaling inputs based on their location. We therefore compared the transcriptional profiles between different spatial gene-expression clusters of the tumor. In the renal cell carcinoma sample (patient 24), gene expression varied with tumor architecture: 305 genes were differentially expressed between small and large nests of tumor (Figures S8F–S8H). Major histocompatibility complex (MHC) class I expression was higher within small tumor nests compared with large tumor nests (Figure S8F), suggesting that CD8^+^ T cells within the same tumor may receive different levels of TCR stimulation based on their location within the parenchyma. In the melanoma sample (patient 16), 564 genes were differentially expressed between stromal-adjacent tumor and the remainder of the tumor parenchyma (Figure 6G). Transcript levels of MHC class I and MHC class II were higher in the peripheral, inflammation-adjacent tumor compared with the remainder of the parenchyma, suggesting that TCR stimulation of tumor-specific cells is greatest in this region (Figure 6G). Conversely, in the breast carcinoma sample (patient 24), MHC class I was highly downregulated in capture spots containing tumor, potentially indicating limited tumor-associated antigen presentation to CD8^+^ T cells in this patient (Figure S9G). In the melanoma sample (patient 16), *CXCL9*, *CXCL10*, *CXCL11*, and *CXCL13* as well as tumor growth factor beta (TGF-β) were higher at the tumor periphery, indicating that T cells in this region are subject to a unique chemokine and cytokine milieu compared with those deeper within the tumor (Figure 6G).

Differences in cytokine and chemokine expression between bulk tumor parenchyma and surrounding tissue were also striking (Figure S13A). As examples, *TGFB1* (encoding TGF-β) was enriched in the stroma in three samples, whereas its receptor was more highly expressed predominately in the parenchyma (Figures S13A and S13B). The IFNγ receptor subunit *IFNGR2* was elevated in the parenchyma of four of five tumors (Figures S13A and S13C). *VEGFA* and *VEGFB* were upregulated in the parenchyma all five tumors (Figures S13A and S13D). To catalog signaling networks between specific regions of the BrM microenvironment, we used CellPhoneDB^52^ to interrogate expression of receptor-ligand pairs between among spatial-transcriptomics clusters (Figure S14). This analysis revealed several signaling molecules involved in multiple signaling pathways within the BrM microenvironment, including vascular endothelial growth factor A (VEGF-A), VEGF-B, TGF-β, galectin-9, epidermal growth factor receptor (EGFR), and others (Figures S14A–S14D). Confirming the observations above, CellphoneDB analysis showed that tumor-surrounding inflammatory stroma expressed *TGFB1* and *TGFB3*, with its corresponding receptors found in the tumor parenchyma of patient 16 (Figure S14E). In patient 24, where gene expression varied by tumor histology (Figure S10), TGF-β signaling also varied within different tumor regions (Figure S14F). Certain galectin-9 signaling pathways also varied between tumor regions (Figures S14E and S14F). Given the differential localization of phenotypically and clonally restricted CD8^+^ T cell subsets in the tumor, our results together indicate that antigen signaling (or lack thereof) localizes these distinct CD8^+^ T cell subsets to specific niches within BrMs, where they receive markedly different signaling inputs. Additionally, the commonality of some of these signaling pathways across different patients (Figures S14A–S14D) within our cohort indicates the presence of targetable signaling pathways across BrMs of different primary histologies.

## Discussion

In this work, we describe the CD8^+^ T cell infiltrate of human BrMs. The BBB, which maintains the unique immune-privileged environment of the brain, appears to break down in BrMs. The resulting BTB is more permeable but maintains some features of the BBB and varies with tumor type.^2^ Although BrMs show some response to ICB, the semi-privileged immune environment created by the BTB could, in theory, restrict entry and/or maintenance of immune cells into the BrM TME. Here, we show that human BrMs are well infiltrated by T cells, although the degree of infiltration varies among patients.

We find that distinct CD8^+^ T cell subsets populate BrMs compared with patient-matched blood. Most CD8^+^ T cells within the tumor are PD-1^+^, and our scRNA-seq analyses clustered these PD-1^+^ cells into dividing and terminally differentiated cells—which are clonally related—and two memory-like subsets of cells that share TCR overlap with each other but not with dividing or terminally differentiated cells. Overall, TCR overlap is low between tumor-infiltrating and circulating PD-1^+^ CD8^+^ T cells, consistent with the absence of circulating tumor-specific CD8^+^ T cells in HPV+ head and neck cancer patients.^30^ However, memory-like tumor-infiltrating cells do have meaningful clonal overlap with circulating cells. Notably, we find that BrMs contain non-tumor-specific CD8^+^ T cells. These bystander cells express markers also found on exhausted progenitor populations and are found in similar frequencies in both tumor-infiltrating and circulating PD-1^+^ CD8^+^ T cells. Finally, we developed a spatial TCR-sequencing technique^42^ to link these phenotypically and clonally restricted CD8^+^ T cells to discrete spatial preferences within the TME. CD8^+^ T cell clones linked to exhaustion are enriched within the tumor parenchyma, where the local cytokine and chemokine milieu varies dramatically from the surrounding stroma, where less-exhausted CD8^+^ T cell clones are found. Based on these findings, our data support a model in which antigen-experienced CD8^+^ T cells infiltrate the TME of BrM in an antigen-independent manner.^31^ Once CD8^+^ T cells are retained in the tumor, antigen signaling, or lack thereof, retains CD8^+^ T cells within distinct spatial niches within the TME. Within each TME niche, the CD8^+^ T cell phenotype evolves with inputs from the surrounding cytokine milieu and, depending on antigen specificity, continued TCR stimulation.

Supporting this model, we show that memory-like BrM-infiltrating CD8^+^ T clones are preferentially retained in the stroma surrounding the tumor parenchyma. In contrast, the terminally differentiated population is predominantly located within the tumor parenchyma itself. Given the limited clonal and phenotypic overlap between bystander cells and the terminally differentiated population, it is likely that antigen stimulation retains terminally differentiated PD-1^+^ CD8^+^ T cells in the tumor parenchyma. Within the tumor parenchyma, these cells receive distinct signaling inputs compared with CD8^+^ T cells within the stroma, likely promoting their acquisition of the terminally differentiated phenotype. We show that VEGF expression, for example, is enriched in the tumor parenchyma compared with the stroma; VEGF signaling has been found to promote PD-1, CTLA-4, TIM-3, and TOX expression on CD8^+^ T cells.^53^ This is consistent with the higher expression of these molecules that we observed on terminally differentiated CD8^+^ T cells.

One key question our work addresses is the role of TCF-1^+^ CD8^+^ T cells in human BrMs. TCF-1 is expressed on antigen-specific exhausted progenitor CD8^+^ T cells in mouse models of cancer and chronic infection,^18^^,^^35^ and TCF-1 has been used as a marker of exhausted progenitor CD8^+^ T cells in tumor immunology studies.^27^, ^28^, ^29^ However, TCF-1 is also expressed on many other subsets of CD8^+^ T cells, such as naive and memory cells.^54^ We find that a minority of TCF-1^+^ CD8^+^ T cells within BrMs co-express TOX and PD-1, two proteins also expressed on exhausted, tumor-specific CD8^+^ T cells.^37^, ^38^, ^39^^,^^46^, ^47^, ^48^ Crucially, we show that bystander cells specific for microbial antigens infiltrate BrMs, and a subset of these cells share phenotypic characteristics—such as TCF-1 expression—with exhausted progenitor CD8^+^ T cells. Bystander clones are present at similar frequencies in circulating and BrM-infiltrating PD-1^+^ CD8^+^ T cells. Based on these data, we propose that many of the TCF-1^+^ PD-1^+^ CD8^+^ T cells in the BrM TME are bystanders. These cells may be recruited to the tumor due to increased expression of cytokines and pro-inflammatory signaling molecules rather than through an antigen-driven process. As such, an increased density of TCF-1^+^ CD8^+^ T cells in the BrM TME may indicate a more inflamed tumor rather than a large population of tumor-specific exhausted progenitor CD8^+^ T cells.

The dividing CD8^+^ T cell metacluster identified in our scRNA-seq data could represent an intermediate differentiation state between tumor-specific TCF-1^+^ exhausted progenitor and terminally differentiated cells. These dividing cells in human BrMs share a gene signature with transitory antigen-specific CD8^+^ T cells that are an intermediate differentiation state between lymphoid-resident exhausted progenitor cells and non-lymphoid-resident, terminally differentiated cells in the LCMV mouse model of T cell exhaustion.^19^^,^^21^ Work in mouse tumor models has shown that tumor-specific exhausted progenitor CD8^+^ T cells are present in tumor-draining lymph nodes and that they are clonally related to tumor-infiltrating, terminally differentiated CD8^+^ T cells.^33^, ^34^, ^35^ Further, the maintenance of antigen-specific CD8^+^ T cells within mouse models requires the migration of lymph-node-resident progenitor cells to the tumor; intratumoral TCF-1^+^ tumor-specific cells are not a self-sustaining population.^34^ In the case of metastatic cancer, there may be numerous anatomic sites of tumor-draining lymph nodes depending on the burden of disease, all of which could contain tumor-specific exhausted progenitor CD8^+^ T cells. A lymphatic system draining the brain has recently been described^55^, ^56^, ^57^ and suggests that cervical lymph nodes could also serve as a reservoir of BrM-specific progenitor CD8^+^ T cells. Our histology experiments also revealed peritumoral inflammatory tissue in some tumors, and other groups have demonstrated tertiary lymphoid structures within the TME,^58^, ^59^, ^60^ both of which could harbor TCF-1^+^ tumor-specific exhausted progenitor cells. While our data do not preclude the presence of a small population of tumor-specific progenitor exhausted CD8^+^ T cells within the BrM TME, we suggest that these other sites may be an important reservoir of tumor-specific exhausted progenitors. This is consistent with a recent report^31^ in metastatic melanoma but differs from work in HPV+ head and neck cancers that identified tumor-infiltrating, tumor-specific progenitor exhausted CD8^+^ T cells.^30^ However, this discrepancy is likely due to the lymphoid tissues—such as tonsils—in which HPV+ head and neck cancer arises.^61^

Our findings have a number of therapeutic implications. First, the dense infiltration of BrMs by CD8^+^ T cells provides a rational basis for the continued use and further development of immunotherapies in the BrM setting. Next, our data support the use and continued investigation of combination therapies with PD-1 pathway blockade to enhance rescue of exhausted, tumor-specific CD8^+^ T cells by targeting additional inhibitory molecules expressed on BrM-infiltrating terminally differentiated CD8^+^ T cells, such as CTLA-4 and LAG3.^62^^,^^63^ This is consistent with clinical data showing that patients with BrM benefit from immunotherapies,^64^^,^^65^ particularly dual checkpoint inhibition.^62^ Nonetheless, progression in the brain remains a barrier to disease control and long-term survival in these patients, demonstrating that current combination approaches alone are insufficient. Given the toxicity associated with dual checkpoint blockade, we suggest that the surface molecules we identify on metacluster A/D CD8^+^ T cells may be preferentially expressed on tumor-specific cells and could be investigated for putative checkpoint function. Finally, our results suggest an additional therapeutic strategy: targeting the unique signaling niches in which BrM-infiltrating CD8^+^ T cells reside. Such an approach, which harnesses the immune system locally within the BrM TME, may prove to be less toxic and more durable in promoting disease control in the brain. Together, our findings support the continued development of immunotherapeutic strategies that harness the anti-tumor efficacy of BrM-infiltrating CD8^+^ T cells.

### Limitations of the study

Among the BrMs we analyzed, intracranial progression while on systemic therapy appeared to be associated with reduced BrM lymphocytic infiltration. Although all patients in our cohort were immunotherapy naïve, it is possible that their current treatment or treatment histories—such as the type of systemic therapy administered during previous definitive treatment or a history of radiation therapy—may have impacted the phenotypes of CD8^+^ T cells infiltrating recurrent or progressive BrM. It should also be noted that BrMs are often found early in patients with an active cancer diagnosis before they are large enough to cause symptoms or require surgical resection. As this was not the case for our patient cohort, the CD8^+^ T cell phenotypes we observed could be more specific to advanced BrMs in which tumor-infiltrating lymphocytes may be resident in the TME for months before diagnosis and treatment.

While we show that our cohort of 31 BrMs are well infiltrated by CD8^+^ T cells, some assays—particularly scRNA-seq and spatial TCR-sequencing—were performed with a limited sample size. In particular, we provide spatial TCR information for only two patients. Validation of the spatial segregation of exhausted and memory-like CD8^+^ T cell clones within the BrM TME will require these assays to be performed with a larger sample size. Additionally, we do not identify tumor-specific cells in this study. Based on prior studies and the enrichment of microbial-specific cells in metaclusters B and C, it is likely that metacluster A/D cells are tumor specific, but future work should precisely identify the phenotype of tumor-specific cells in BrMs.

## STAR★Methods

### Key resources table

**Table:** 

REAGENT or RESOURCE	SOURCE	IDENTIFIER
**Antibodies**		

Tim3 BV421	BD	Cat# 565562; RRID: AB_2744369
HLA-DR Pacific Blue	BioLegend	Cat# 307633; RRID: AB_1595444
CD8 BV510	BioLegend	Cat# 301048; RRID: AB_2561942
CD39 BV605	BioLegend	Cat# 328236; RRID: AB_2750430
CD69 BV650	BioLegend	Cat# 310934; RRID: AB_2563158
CD45RA BV711	BioLegend	Cat# 304138; RRID: AB_2563815
PD-1 BV786	BioLegend	Cat# 329930; RRID: AB_2563443
Tcf-1 (rabbit), unconjugated	Cell Signaling Technologies	Cat# 2203S
CD45 Spark Blue 550	BioLegend	Cat# 368549; RRID: AB_2820024
CD38 BB700	BD	Cat# 566445; RRID: AB_2744375
TIGIT PerCP/eFluor710	Invitrogen	Cat# 46-9500-41; RRID: AB_10853679
Granzyme B Biotin	Mabtech	Cat# 3485-6-250; RRID: AB_907253
KI-67 BB790	BD	Custom
TOX PE	Invitrogen	Cat# 12-6502-82; RRID: AB_10855034
CTLA-4 PE/Dazzle594	BioLegend	Cat# 349922; RRID: AB_2566198
FOXP3 PE/Cy5	Invitrogen	Cat# 15-4776-42; RRID: AB_1963595
CD28 PE/Cy7	BioLegend	Cat# 302926; RRID: AB_10644005
CD127 APC	BioLegend	Cat# 351316; RRID: AB_10900804
CCR7 Spark NIR 685	BioLegend	Cat# 353258; RRID: AB_2860926
CD3 R718	BD	Cat# 751978
CD4 APC/Fire810	BioLegend	Cat# 344662; RRID: AB_2860884
anti-rabbit AF488	Thermo Fisher	Cat# A-11008; RRID: AB_143165
Streptavidin BB755	BD	Custom
TotalSeq-C0251 anti-human Hashtag 1 Antibody	BioLegend	Cat# 394661; RRID: AB_2801031
TotalSeq-C0252 anti-human Hashtag 2 Antibody	BioLegend	Cat# 394663; RRID: AB_2801032
Pan-Cytokeratin Alexa Fluor 594 (for immunofluoresence)	BioLegend	Cat# 628606; RRID: AB_2566437

**Chemicals, peptides, and recombinant proteins**		

CEFX Ultra SuperStim Pool	JPT	Cat# PM-CEFX-2
Zombie NIR	Biolegend	Cat# 423106
DAPI	Thermo Fisher	Cat# 62248

**Critical commercial assays**		

IFNγ Secretion Assay – Detection Kits, human	Miltenyi	Cat# 130-054-202
TCR profiling (of circulating CD8^+^ T cells)	Adaptive Biotechnologies	Human TCRB immunoSEQ
Visium Spatial Gene Expression Reagent Kit	10X Genomics	Cat# 1000184
Library Construction Kit	10X Genomics	Cat# 1000190

**Deposited data**		

scRNA-seq data	This paper	GEO: GSE179373
Spatial transcriptomics data	This paper	GEO: GSE179572
Spatial TCR-sequencing reads	This paper	BioProject: PRJNA742564
VDJdb TCR sequences	VDJdb	https://vdjdb.cdr3.net/
Processed scRNA-seq and CellphoneDB analysis	This paper	Mendeley data: https://doi.org/10.17632/tdggxgygrw.1

**Oligonucleotides**		

Dual Index Kit TT Set A	10X Genomics	Cat# 1000215

**Software and Algorithms**		

Cell Ranger, version 4	10X Genomics	https://support.10xgenomics.com/single-cell-gene-expression/software/pipelines/latest/what-is-cell-ranger
Space Ranger, version 1	10X Genomics	https://support.10xgenomics.com/spatial-gene-expression/software/pipelines/latest/what-is-space-ranger
Loupe Browser, version 5	10X Genomics	https://support.10xgenomics.com/single-cell-gene-expression/software/visualization/latest/what-is-loupe-cell-browser
MiXCR	Bolotin et al., *Nature Methods* 2015	https://github.com/milaboratory/mixcr/
Seurat	Stuart et al., *Cell* 2019	CRAN
Spatial TCR sequencing analysis	This paper	https://github.com/whhudson/spatialTCR (https://doi.org/10.5281/zenodo.6368907)
FlowJo	BD Biosciences	https://www.flowjo.com/

### Resource availability

#### Lead contact

Further information and requests for resources and reagents should be directed to and will be fulfilled by the lead contact, William Hudson (william.hudson@emory.edu).

#### Materials availability

This study did not generate new unique reagents.

### Experimental model and subject details

#### Human samples

All brain metastases from immunotherapy-naïve patients resected at Emory University Hospital during the collection period were included unless consent was not obtained, there was insufficient tissue, or in some cases the surgery was performed emergently after hours. Blood samples were collected during surgery in BD Vacutainer lithium heparin tubes. Tumor and blood samples were held at 4 °C until retrieved for processing, typically within 1 hour after resection. Nearly all patients with surgically-resected brain metastases are treated with the immunosuppressive glucocorticoid dexamethasone prior to surgery. Standard dexamethasone administration was a 10 mg loading dose followed by 4 mg every 6 hours until surgery. The duration of dexamethasone therapy prior to surgery was not correlated with CD45^+^ lymphocyte or CD8^+^ T cell infiltration of brain metastases in our cohort (Figures S1E and S1F).

Experiments were carried out with the approval of the Emory University Institutional Review Board under protocols IRB00045732, IRB00095411, and STUDY00001995.

### Method details

#### Tissue processing and cell extraction

Tumors were weighed, cut into small pieces, and then incubated in Leibovitz media with collagenase I, II, and IV, elastase and DNAse for 60 minutes, shaking at 37°C. Tissue and media were then passed through a 70 μm single-cell strainer and cells were pelleted by centrifugation. The pellet was resuspended in 44% Percoll, underlaid with 67% Percoll, and centrifuged. Immune cells were collected from the gradient interface and washed with 2% FBS in PBS. Washed cells were resuspended in 1–2 mL of PBS containing 2% FBS. 10 μL of cells were stained for 20 minutes with anti-CD45 and anti-CD8 antibodies (and later, anti-CD4 and CD19 antibodies). CountBright counting beads (Thermo Fisher) were added to stained cells and the sample was analyzed on a BD LSR II flow cytometer to determine the absolute number of cell populations. For isolation of circulating immune cells, a lymphocyte separation medium (Corning catalog #25-072-CV) gradient was performed according to the manufacturer’s instructions. With the exception of scRNA-seq samples (see below), cells were then frozen at −80°C in 10% DMSO in FBS and subsequently transferred to liquid nitrogen for long-term storage. For scRNA-seq of PD-1^+^ CD8^+^ T cells shown in Figure 3 and related experiments, cells were immediately stained with antibodies after isolation for the sort as described below. For scRNA-seq validation of spatial TCR methods, an additional capture of CD4^+^ and CD8^+^ T cells were isolated from frozen cells of patients 16, 26, and 27; additional frozen cells of patient 15 were not available.

#### Flow cytometry

For scRNA-seq and TCR sequencing, freshly-isolated tumor-infiltrating and circulating immune cells were stained with extracellular antibodies for 30 minutes, washed with 2% FBS in PBS and sorted on a BD FACS ARIA II in the Emory University School of Medicine Flow Cytometry Core. Tumor-infiltrating PD-1^+^ and matched circulating naïve CD8^+^ T cells were submitted to the Emory Yerkes NHP Genomics Core where gene expression and TCR sequence libraries were generated with a 10X Genomics Chromium controller. DNA was extracted from circulating PD-1^+^ and PD-1^-^ CD8^+^ T cells using the All-Prep DNA/RNA Micro Kit from Qiagen and sent to Adaptive Biotechnologies for survey-level TCRβ sequencing.

For high-parameter flow cytometry, frozen cells were quickly thawed in a 37°C water bath, washed with pre-warmed (37°C) 10% FBS in RPMI, and resuspended in staining buffer (PBS with 2% FBS). Staining was performed at room temperature. Washed cells were stained first with Zombie NIR viability dye for 30 minutes, then extracellular antibodies were added in BD Horizon Brilliant Stain Buffer for 30 minutes. Cells were washed twice in staining buffer. The eBioscience Foxp3 Transcription Factor Staining Buffer Set was then used for fixation and permeabilization according to the manufacturer’s protocols. Cells were then stained with intracellular antibodies for 30 minutes, washed twice with permeabilization buffer, stained with secondary antibodies for 30 minutes, washed with permeabilization buffer once and staining buffer once, then resuspended in staining buffer for data acquisition. Data was acquired on a four-laser Cytek Aurora flow cytometer in the Winship Pediatrics Flow Cytometry Core.

#### PBMC expansion, CEFX stimulation, and IFNγ capture

PBMCs were quickly thawed, washed, and counted in pre-warmed 10% FBS in RPMI. Cells were expanded as described previously.^30^ Briefly, cells were resuspended in complete CTS media containing CTS OpTmizer (Gibco) with CTS supplement, L-glutamine, Penicillin/Streptomycin, Human AB serum (Sigma), recombinant IL-2, IL-7, and IL-15 (Peprotech), and CEFX Ultra SuperStim peptide pool (JPT). Cells were plated and incubated at 37°C for five days and then split with the above CTS complete media and cytokines, without the re-addition of the CEFX peptides. Five days later, cells were washed and rested overnight at 37°C in complete CTS media without cytokines. The next day, the CEFX Ultra SuperStim peptide pool was added to cells at a final concentration of 2.5 μg/mL. An equal volume of DMSO was added to unstimulated cells. Cells were incubated 5 hours at 37°C . Manufacturer’s protocols were then followed for the Miltenyi cytokine secretion assay using the cytokine catch reagent and cytokine detection antibody (IFNγ PE) to label cells secreting IFNγ. Cells were then stained for viability, CD3, and CD8 and sorted on a BD FACS ARIA II in the Emory University School of Medicine Flow Cytometry Core (Figure S7A). DNA was isolated from sorted IFNγ^+^ and IFNγ^-^ CD8^+^ T cells using the All-Prep DNA/RNA Micro Kit from Qiagen and sent to Adaptive Biotechnologies for survey-level TCRβ sequencing. A clone was considered CEFX-specific if found at least twice in the IFNγ^+^ population and with a frequency ≥5x higher in IFNγ^+^ cells compared to IFNγ^-^ cells^.^

#### Single cell RNA-sequencing and peripheral TCR profiling

Single-cell gene expression and VDJ (paired TCRα/β) libraries were generated by the Emory Yerkes NHP Genomics Core from freshly-isolated tumor-infiltrating PD-1^+^ CD8^+^ T cells and matched naïve circulating CD8^+^ T cells isolated by FACS and mixed at a 10:1 ratio. TILs and naive circulating cells were stained with TotalSeq hashing antibodies (BioLegend) prior to combining for cell capture and library preparation. TCR sequencing (TCRβ only) from sorted circulating cells was performed by Adaptive Biotechnologies.

#### Spatial transcriptomics

Surgically resected tissue was embedded in OCT and immediately flash frozen in a dry ice/2-methylbutane bath. Sections 10 μm thick were placed onto a Visium Gene Expression slide and stored at −80°C for up to one week. Slides were subsequently H&E stained according to the manufacturer’s instructions and imaged with a Lionheart Microscope (Biotek) at 10X magnification. Tissue permeabilization, reverse transcription, second strand synthesis, and cDNA amplification was performed according to the manufacturer’s instructions. 25% of amplified cDNA was used for gene expression library preparation; libraries were sequenced on a NovaSeq 6000 instrument at the Yerkes Nonhuman Primate Genomics Core.

For TCR library preparation, 5 μL of amplified Visium cDNA was used as template in a 35-cycle PCR reaction using 45 previously-described^66^ pooled *TRBV* forward primers and the Illumina read 1 reverse primer. Partial Illumina read 2 sequences (5′- GTGACTGGAGTTCAGACGTGTGCTCTTCCGATCT-3′) were added to the 5′ end of each *TRBV* forward primer. PCR product was purified without fragmentation using SPRIselect beads and quantified using a Qubit 1X dsDNA HS Assay Kit (Thermo Fisher). Sample index PCR was performed with the 10X Genomics Library Construction Kit using primers from the 10X Genomics Dual Index Kit TT Set A according to the manufacturer’s instructions (protocol CG000239, 10X Genomics). Libraries were again bead purified and sequenced on an Illumina MiSeq instrument at the Yerkes Nonhuman Primate Genomics Core.

### Quantification and statistical analysis

#### Flow cytometry

Flow cytometry were analyzed in FlowJo, using the FlowSOM and UMAP plugins.^67^^,^^68^ Summary graphs and statistics were generated in GraphPad Prism v8.

#### scRNA-seq analysis

Single-cell gene expression data were aligned and TCR sequences determined with CellRanger. Outlier cells with high numbers of reads originating from mitochondrial genes and presumed doublets were excluded from the dataset, and genes encoded on the Y or mitochondrial chromosomes were excluded from gene expression analysis. 22,898 cells passed quality control and were analyzed here. Data were normalized and scaled with the Seurat package in R^69^ and plots made with ggplot2^70^ or GraphPad Prism. Shared nearest neighbor clustering was performed in Seurat with 100 neighbors, 21 principal components, and a resolution of 0.9. UMAP dimensionality reduction was performed with 100 neighbors, 21 principal components, and a minimum distance of 0. Seurat’s BuildClusterTree with identical parameters was used for was used to create a phylogenetic tree of identified clusters. Gene set enrichment analysis (GSEA) was performed with the fgsea package in R,^71^ using sign(fold change)∗-log_10_p_adj_ from Seurat’s FindMarkers function as the ranking statistic. Morisita-Horn indices were calculated with the divo package in R.^72^ Shannon indices were calculated in R with the vegan package.^73^ Genes encoding proteins with transmembrane helices were identified in R with Ensembl annotations accessed via biomaRt.^74^^,^^75^

For TCR analysis, cells or sequencing reads with identical *TRBV* and *TRBJ* gene families and identical TCRβ CDR3 amino acid sequences were considered to originate from same clone. Cells from scRNA-seq with undetermined TCRβ clonotypes but known TCRα sequences were assigned to a clonotype if all other cells with the TCRα were paired with a single TCRβ clone. To search for cells with known antigen specificity, we queried the VDJdb^76^ (accessed February 2021) with paired TCRα/β sequences from our scRNA-seq data, requiring identical TCRα and TCRβ CDR3 sequences as well as exact matches for *TRAV, TRAJ, TRBV, TRBJ* genes to be considered a T cell with known antigen specificity. This resulted in the identification of two clones specific for the CMV protein IE1.

#### Spatial transcriptomics analysis

Space Ranger was used for sequence analysis and alignment. Loupe Browser was to visualize data for pathology review; tissue regions were called by graph-based clustering and annotated within Loupe Browser. Detailed analysis and visualization were conducted in R with the Seurat package.^69^ Genes were considered below the limit of detection if expressed below 10 counts or were expressed in two or fewer spots. For analyses of boundary and tumor gene expression (Figure S13) the following clusters were used: sample 16, clusters 5 and 3/4 (boundary and tumor, respectively); sample 19, clusters 3 and 5; sample 24, clusters 4 and 1/2/3; sample 26, clusters 6 and 1/2/3; sample 27, clusters 7 and 2/3/4/5/6.

#### Spatial TCR-sequencing analysis

The MiXCR^77^ analyze pipeline was performed on read 2 sequences, and supporting reads for each clonotype were written with the exportReadsForClones command. The UMI and spatial barcodes were extracted from the paired read for each supporting sequencing read. A detailed protocol for obtaining TCR sequences from spatial transcriptomics data is available in an accompanying manuscript^42^.

## Data Availability

• Single-cell RNA-seq data and spatial transcriptomics have been deposited at GEO and are publicly available as of the date of publication. Spatial TCR-seq reads have been deposited in the SRA. Microscopy images from spatial transcriptomics are publicly available in the GEO deposition. Accession numbers and DOIs are listed in the key resources table. • Code used to identify and map TCR sequences from spatial transcriptomics data has been deposited on Github. The DOI is listed in the key resources table. • Any additional information required to reanalyze the data reported in this paper is available from the lead contact upon request.

## Supplemental information

Document S1. Figures S1–S14

Document S2. Article plus supplemental information
