# Supplementary material for: Distinct phenotypic states and spatial distribution of CD8+ T cell clonotypes in human brain metastases
Source: Cell Rep Med. 2022 Apr 27;3(5):100620. doi: 10.1016/j.xcrm.2022.100620 (PMC9133402; doi:10.1016/j.xcrm.2022.100620)
Supplement: Document S1. Figures S1–S14 [file mmc1.pdf]

Cell Reports Medicine, Volume *3*

Supplemental information

Distinct phenotypic states and spatial distribution of CD8$^+$ T cell clonotypes in human brain metastases

Lisa J. Sudmeier, Kimberly B. Hoang, Edjah K. Nduom, Andreas Wieland, Stewart G. Neill, Matthew J. Schniederjan, Suresh S. Ramalingam, Jeffrey J. Olson, Rafi Ahmed, and William H. Hudson

A

Primary Tumor Histology	n
Lung carcinoma (4 SCLC, 10 NSCLC)	14
Breast carcinoma	6
Melanoma	4
Poorly differentiated carcinoma, unspecified primary	2
Esophageal carcinoma	1
Adenoid cystic carcinoma	1
Uterine carcinoma	1
Renal cell carcinoma	1
Urothelial carcinoma	1

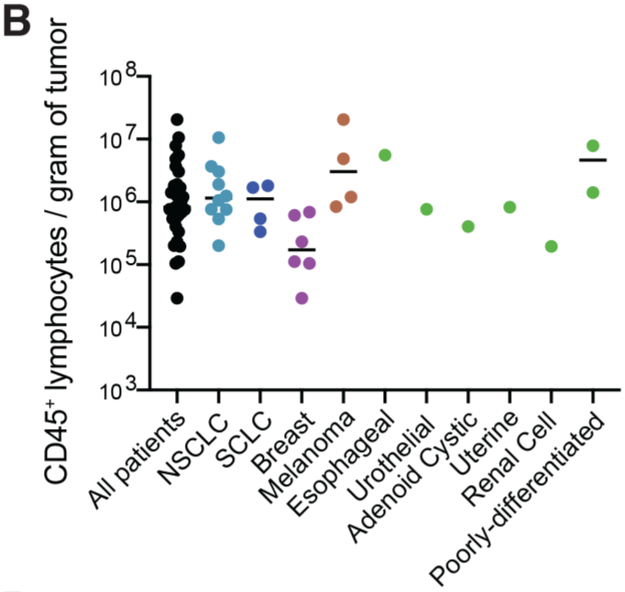

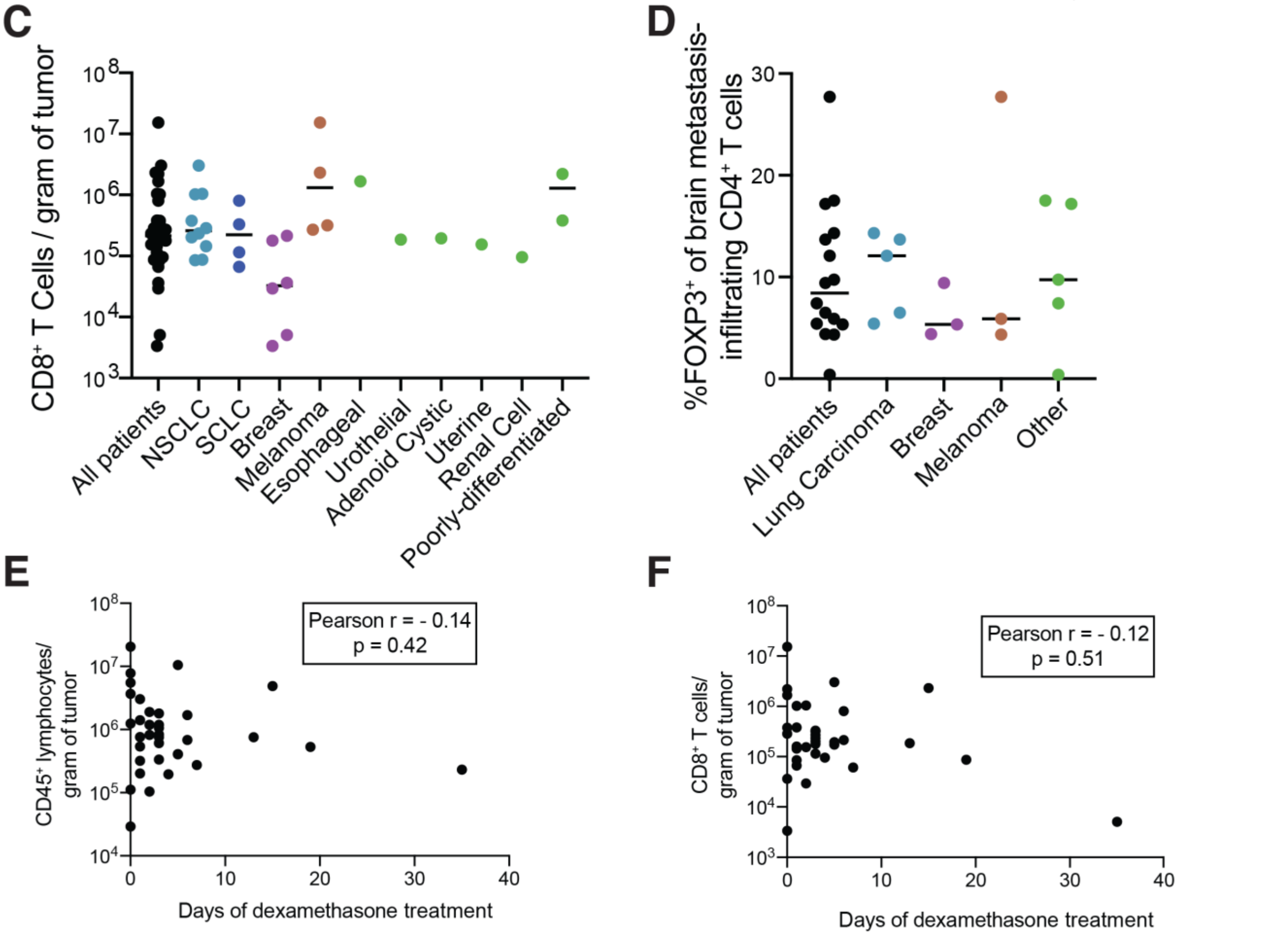

Figure S1: Quantification of lymphocyte infiltration by tumor type and length of dexamethason treatment, related to Figure 1. (A) Primary tumor histology of patients in this study. (B) CD45$^+$ lymphocytes and (C) CD8$^+$ T cells per gram of tumor by individual tumor type for all 31 samples; NSCLC – non-small cell lung cancer, SCLC – small cell lung cancer. (D) Frequency of FOXP3$^+$ cells among brain metastasis-infiltrating CD4$^+$ T cells from 16 patients. Bars indicate median. (E-F) Days of treatment with dexamethasone versus immune cell infiltration are shown for CD45$^+$ lymphocytes (E) and CD8$^+$ T cells (F) for all 31 patients.

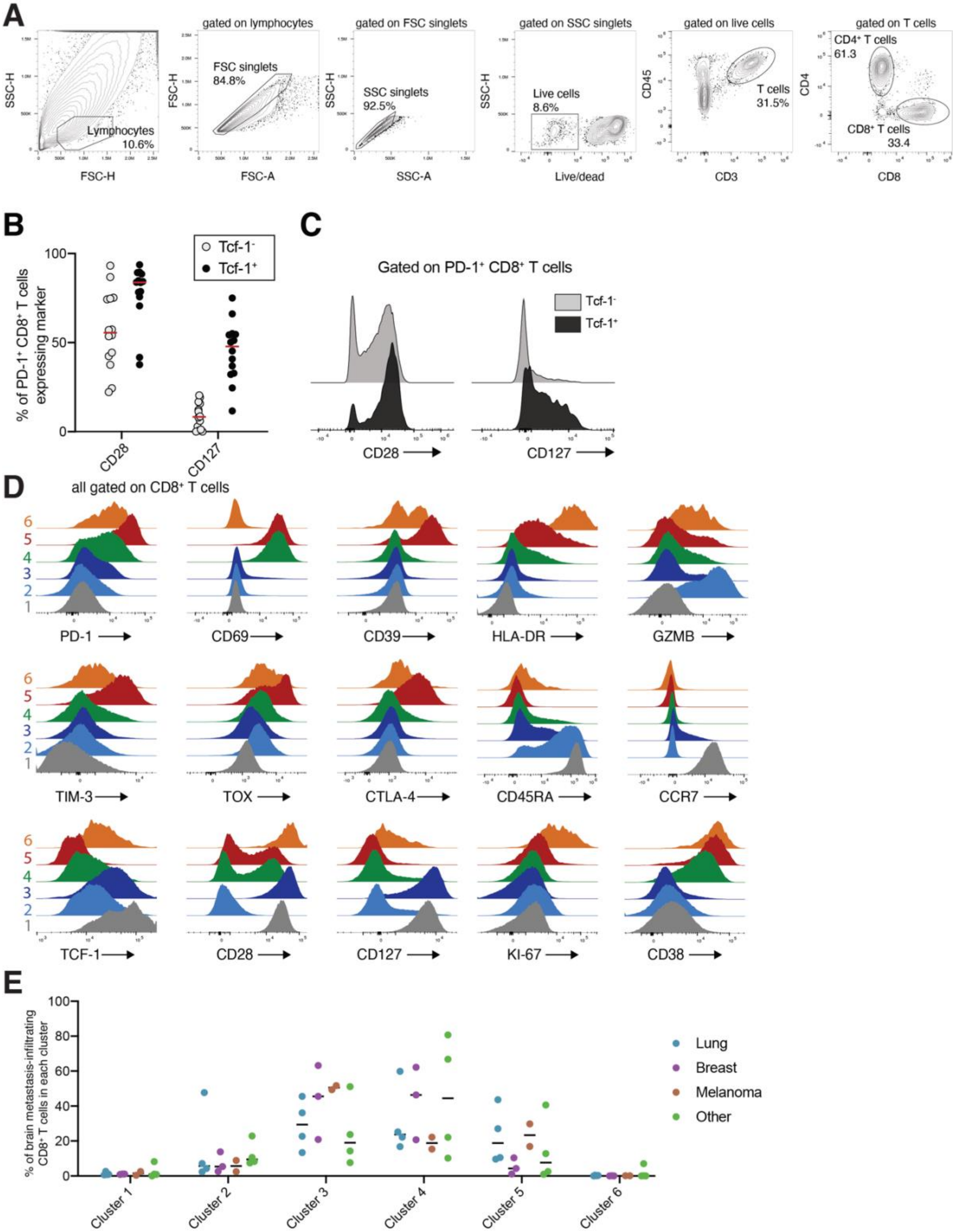

Figure S2: Flow cytometry analysis of brain metastasis-infiltrating CD8⁺ T cells, related to Figure 2. (A) Gating strategy for CD8⁺ T cells. (B) Comparison of CD28 and CD127 expression on TCF-1⁻ and TCF-1⁺ PD-1⁺ CD8⁺ T cells infiltrating brain metastases from 14 patients. Bars on graph indicate medians. (C) Representative flow plots of each marker in (B). (D) Expression of all markers used for UMAP in each CD8⁺ T cell cluster (average among all patients). (E) Percent of brain metastasis-infiltrating CD8⁺ T cells from each of the 13 samples in all FlowSOM clusters by tumor type.

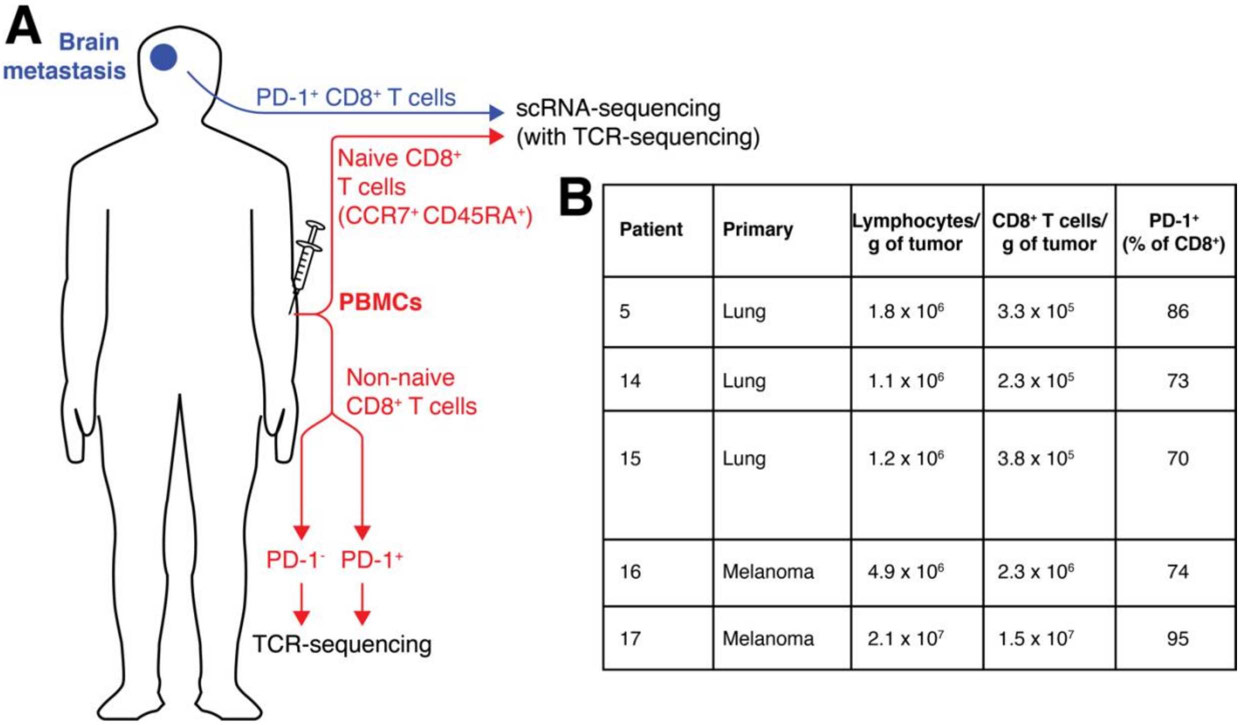

B

Patient	Primary	Lymphocytes/ g of tumor	$CD8^+$ T cells/ g of tumor	$PD\text{-}1^+$ (% of $CD8^+$)
5	Lung	1.8×10^6	3.3×10^5	86
14	Lung	1.1×10^6	2.3×10^5	73
15	Lung	1.2×10^6	3.8×10^5	70
16	Melanoma	4.9×10^6	2.3×10^6	74
17	Melanoma	2.1×10^7	1.5×10^7	95

Figure S3: **Schematic and patient data for single cell RNA-sequencing experiments, related to Figure 3.** (A) PD-1^+ $CD8^+$ T cells were isolated from brain metastases by FACS immediately after surgical resection. These were mixed with naïve $CD8^+$ T cells from peripheral blood after staining with hashing CITE-seq antibodies and subjected to scRNA-seq. Non-naïve $CD8^+$ T cells from blood were sorted into PD-1^- and PD-1^+ populations and separately subjected to TCRβ sequencing. (B) Patient data for scRNA-seq samples. All lung primaries are non-small cell lung tumors.

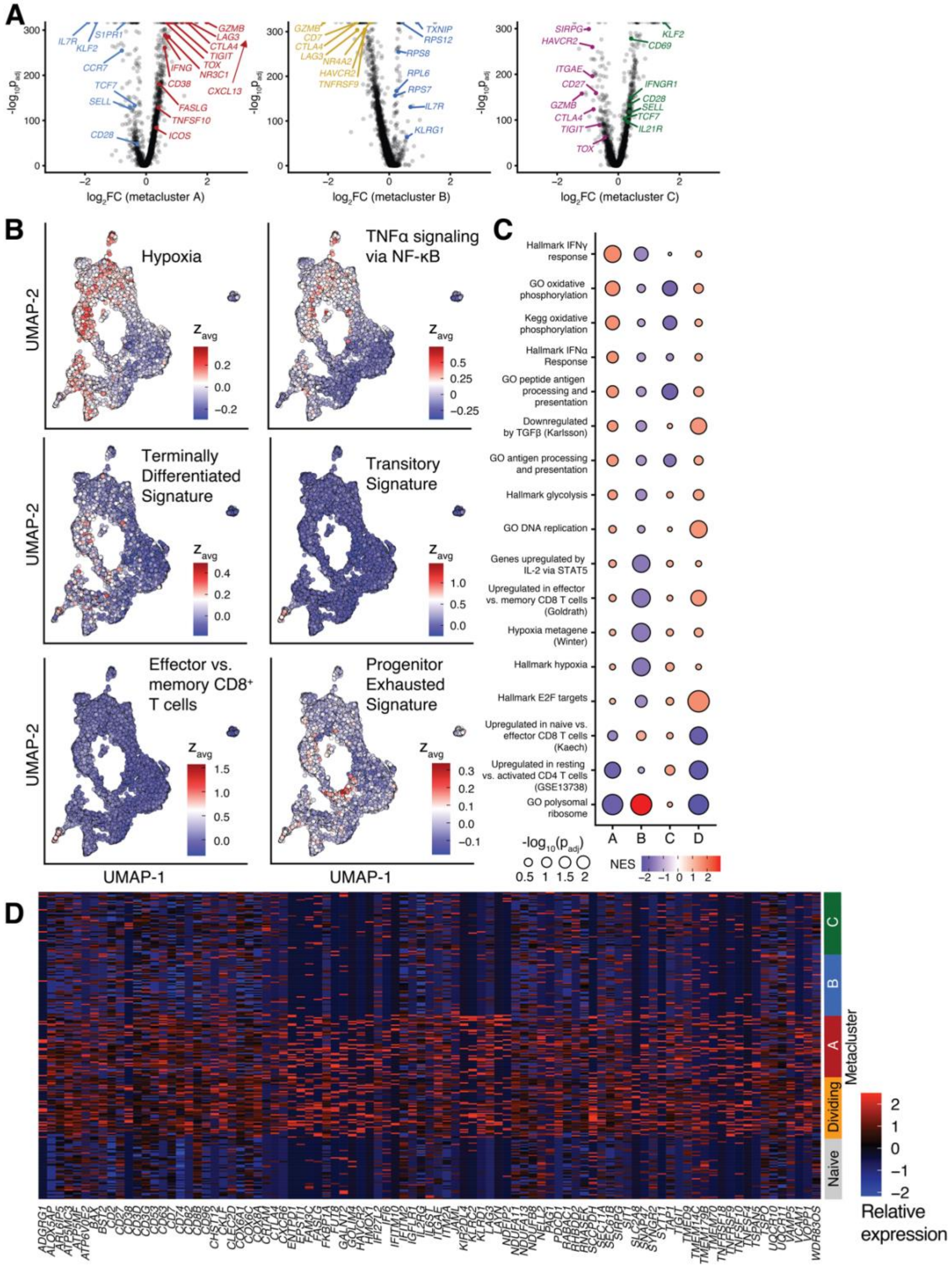

Figure S4: The transcriptional signature of transitory cells from the murine LCMV system is enriched in the Dividing metaclusters, related to Figure 3. (A) Volcano plots showing selected highly differentially-expressed genes in each metacluster compared with all other non-naïve metaclusters. (B) Expression of selected gene sets in each cell, projected on the UMAP. The terminally differentiated, transitory, effector, memory, and progenitor exhausted gene sets are based on the transcriptional phenotype of CD8⁺ T cells in the murine LCMV model (Hudson et al., *Immunity* 2019). Cells are colored by the median z-score of genes in the indicated gene set. (C) Net enrichment score and statistical significance of selected gene sets in each cluster. (D) Expression of surface proteins enriched in CD8⁺ T cells in metacluster A. Each gene showed significant differential expression ($\log_2$ fold change > 0, $p_{adj} < 10^{-100}$) in metaclusters A and contained at least one transmembrane helix.

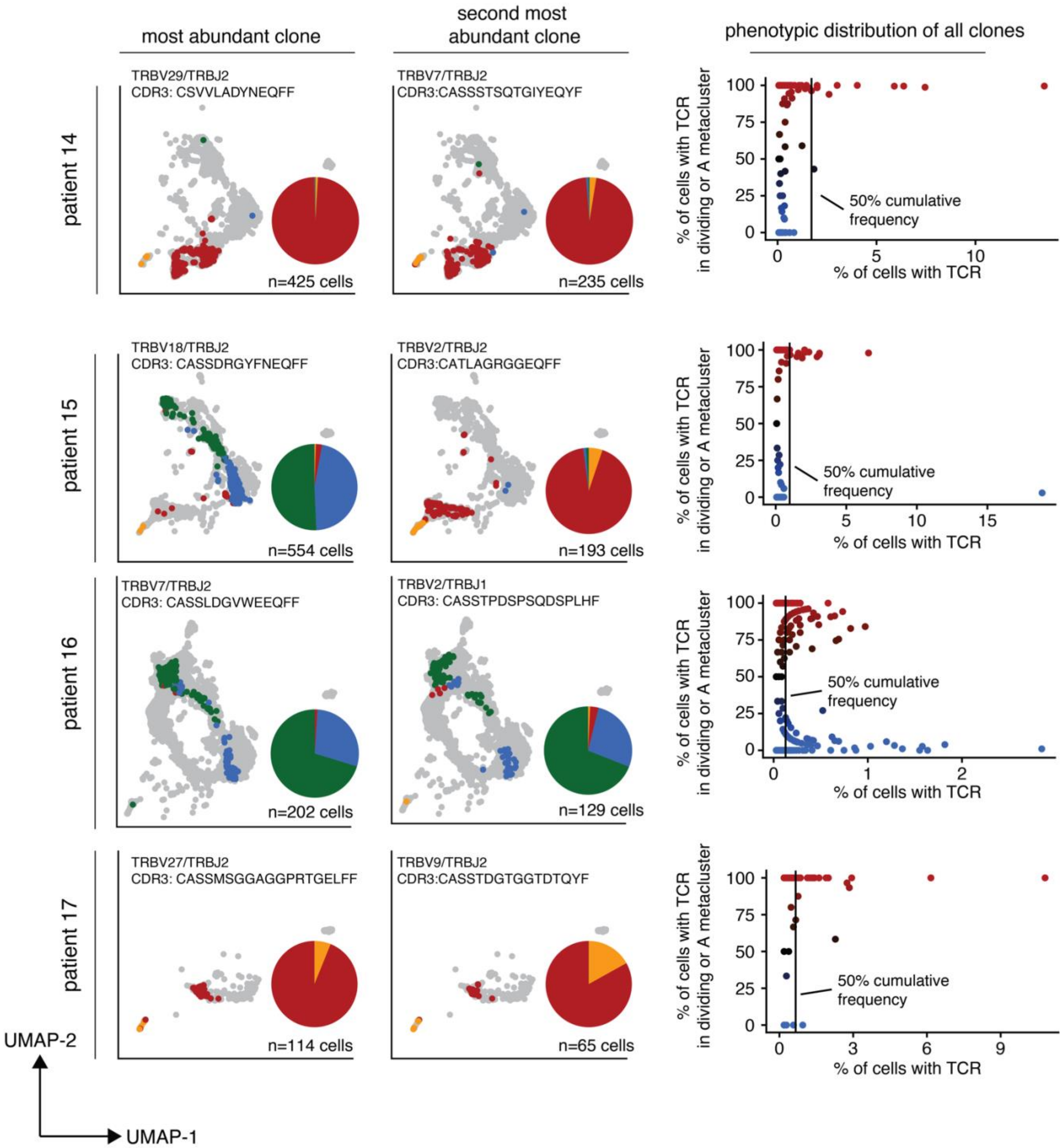

Figure S5: Cells within the most abundant clones of brain metastasis-infiltrating PD-1⁺ CD8⁺ T cells exhibit restriction to a metacluster A/D or B/C phenotype, related to Figure 4. Phenotype of the most abundant (left) and second most abundant (middle) TCR clones from each scRNA-seq sample. Patient 5 is shown in Figure 4F. Cells from other clones of the indicated patient are shown in gray. Inlaid pie charts show distribution of cells by metacluster for the indicated clone. At right, phenotype of all TCR clones detected in each sample. The vertical line indicates the 50% cumulative distribution of clones.

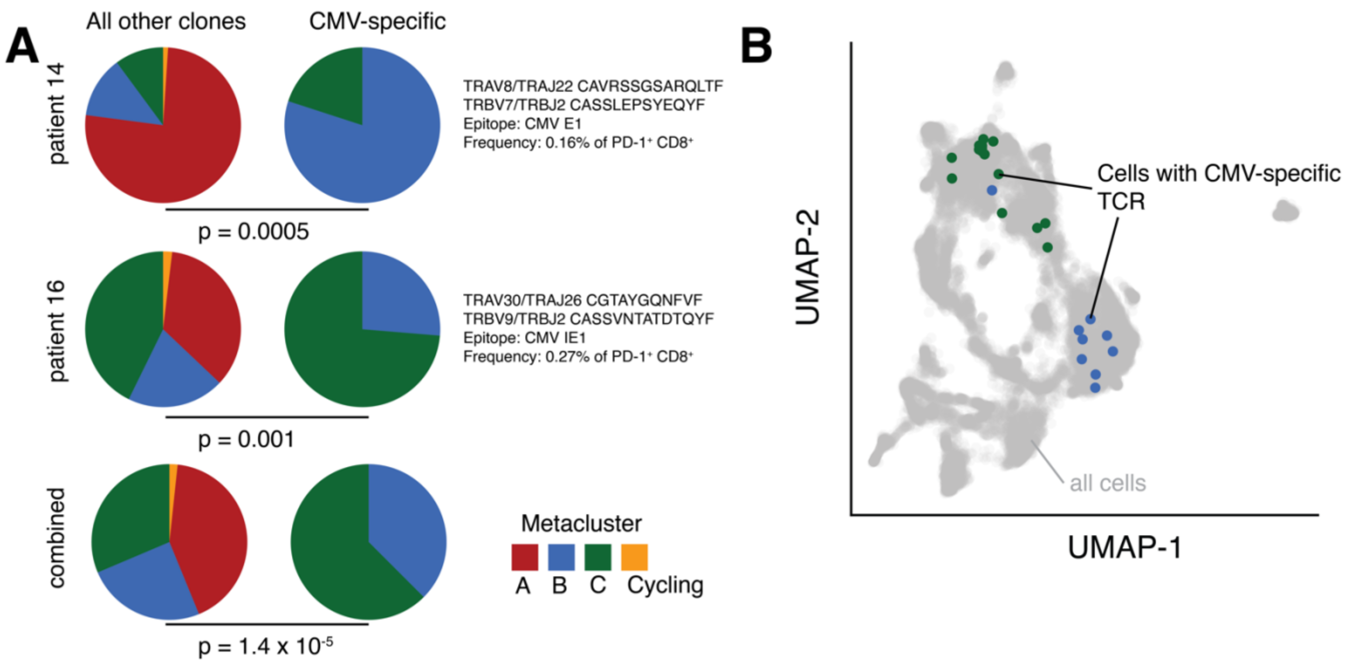

Figure S6: Phenotype of brain metastasis-infiltrating PD-1⁺ CD8⁺ T cells expressing CMV-specific TCRs identified in the VDJdb, related to Figure 5. (A) One CMV-specific TCR was found in patient 14, and one was found in patient 16. Pie charts indicate the scRNA-seq phenotype of CMV-specific clones (right) and all other clones (left) among brain-metastasis PD-1⁺ CD8⁺ T cells. (B) UMAP with brain metastasis-infiltrating PD-1⁺ CD8⁺ T cells expressing a CMV-specific TCR identified in the VDJdb, colored by phenotype. P-values in (A) were calculated with Fisher's exact test.

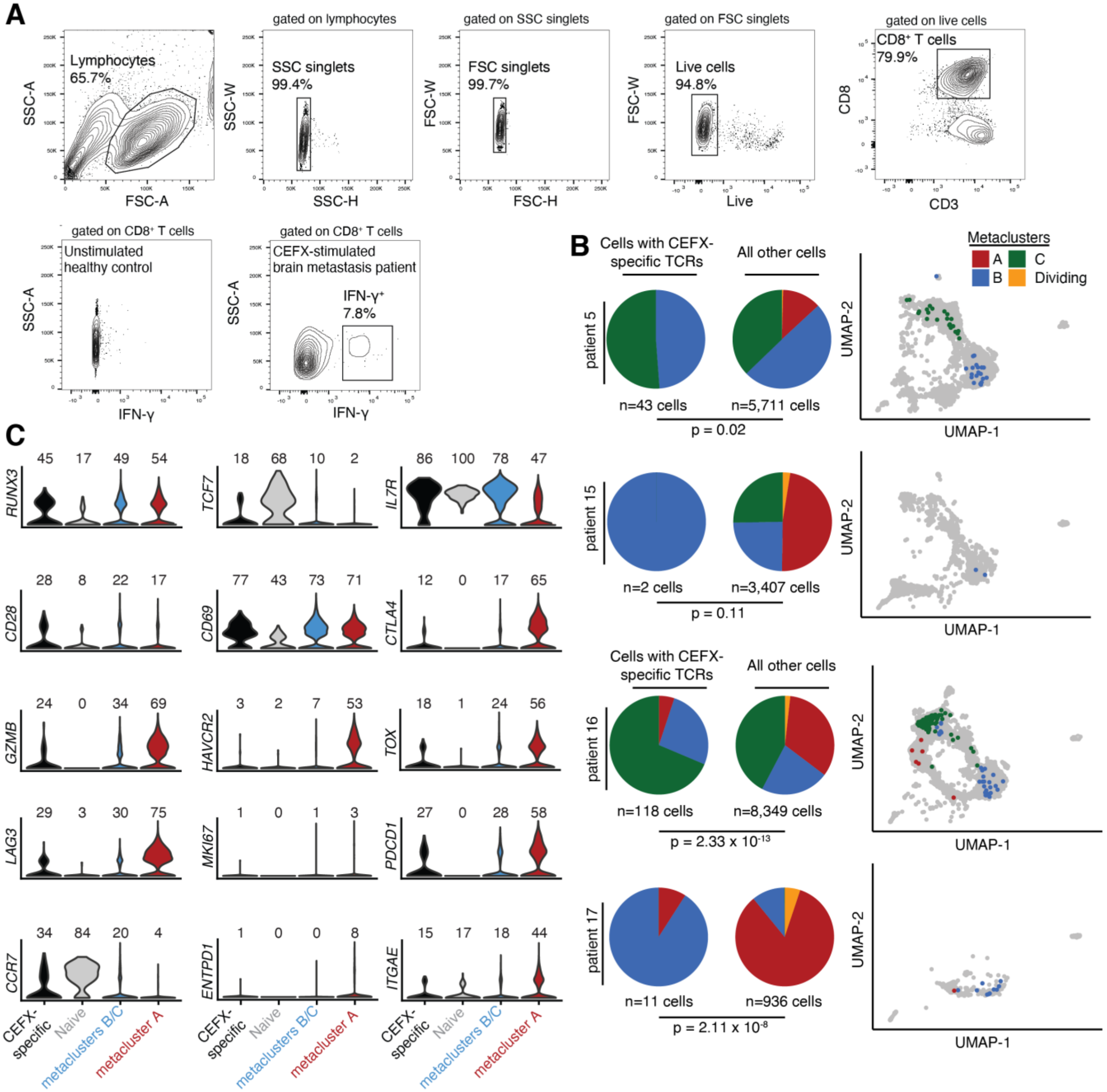

Figure S7: Phenotype of brain metastasis-infiltrating CEFX-specific PD-1⁺ CD8⁺ T cells, related to Figure 5. CEFX-specific CD8⁺ T cells were determined by their expression of TCRs identified in the IFN-γ capture assay shown in Fig. 5. (A) Gating strategy for IFN-γ capture assay to determine CEFX-reactive CD8⁺ T cells. (B) Pie charts show distribution of CEFX-specific (left) and all other (right) brain metastasis-infiltrating PD-1⁺ CD8⁺ T cells by scRNA-seq metacluster. UMAP of brain metastasis-infiltrating PD-1⁺ CD8⁺ T cells from each patient is also shown, with CEFX-specific cells colored by metacluster. All other cells from the patient are shown in gray. (C) Expression of selected genes by CEFX-specific and other brain metastasis-infiltrating PD-1⁺ CD8⁺ T cells. The number above each column indicates percent of cells with measurable expression of each gene. P-values in (B) were calculated with Fisher's Exact Test. PBMCs were not available from patient 14 for this experiment.

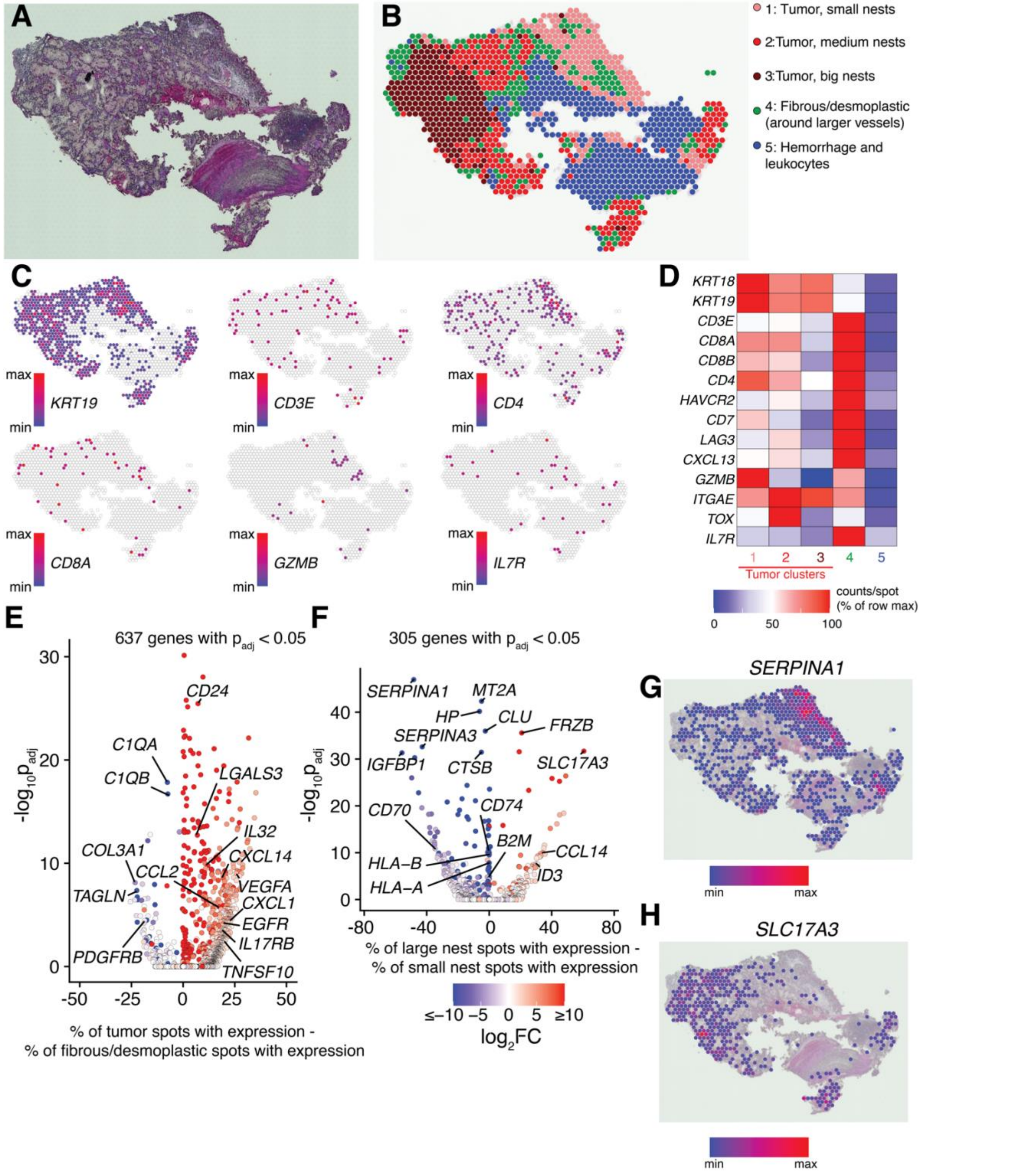

Figure S8: A renal clear cell brain metastasis exhibits transcriptional heterogeneity based on tumor nest size, related to Figure 6. (A) Hematoxylin and eosin-stained (H&E) section of a renal clear cell brain metastasis (patient 24). (B) Graph-based clusters of gene expression with pathologist annotations. (C) Spatial expression of selected genes. (D) Heatmap of normalized gene expression density in each cluster. (E) Differential gene expression between tumor (clusters 1, 2, and 3) and surrounding fibrous/desmoplastic tissue (cluster 4). (F) Differential gene expression between large nests of tumor (cluster 3) and small nests of tumor (cluster 1). (G-H) Expression of two example genes highly expressed in small nests (*SERPINA1*, panel G) and large nests (*SLC17A3*, panel H).

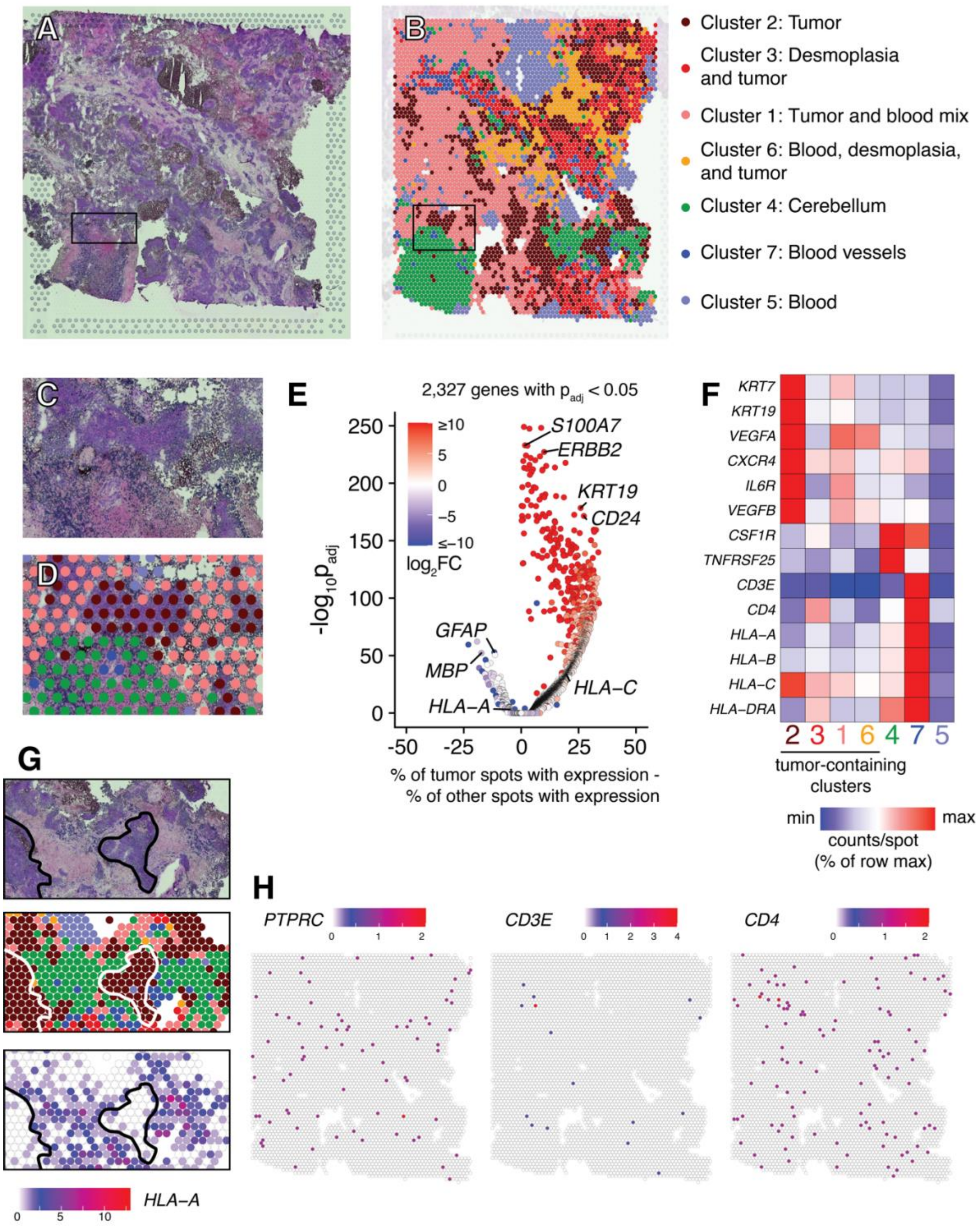

Figure S9: Spatial transcriptomics of a poorly-infiltrated brain metastasis of breast carcinoma reveals spatial heterogeneity of gene expression and loss of MHC I expression by tumor regions, related to Figure 6. (A) H&E-stained section of a breast carcinoma brain metastasis (patient 26). (B) Annotated graph-based clusters of gene expression. (C-D) Inset of H&E staining and clusters. (E) Differential gene expression of tumor-containing regions (clusters 1, 2, 3, and 6) compared to other clusters. (F) Heatmap of normalized gene expression density in each cluster. (G) Loss of *HLA-A* expression in tumor clusters. Top panel: H&E staining; middle: graph-based clusters; bottom panel: expression of *HLA-A*. Selected tumor regions are outlined. (H) Expression of *PTPRC* (encoding CD45), *CD3E*, a pan T cell marker, and *CD4*. *CD8A* expression was below the limit of detection.

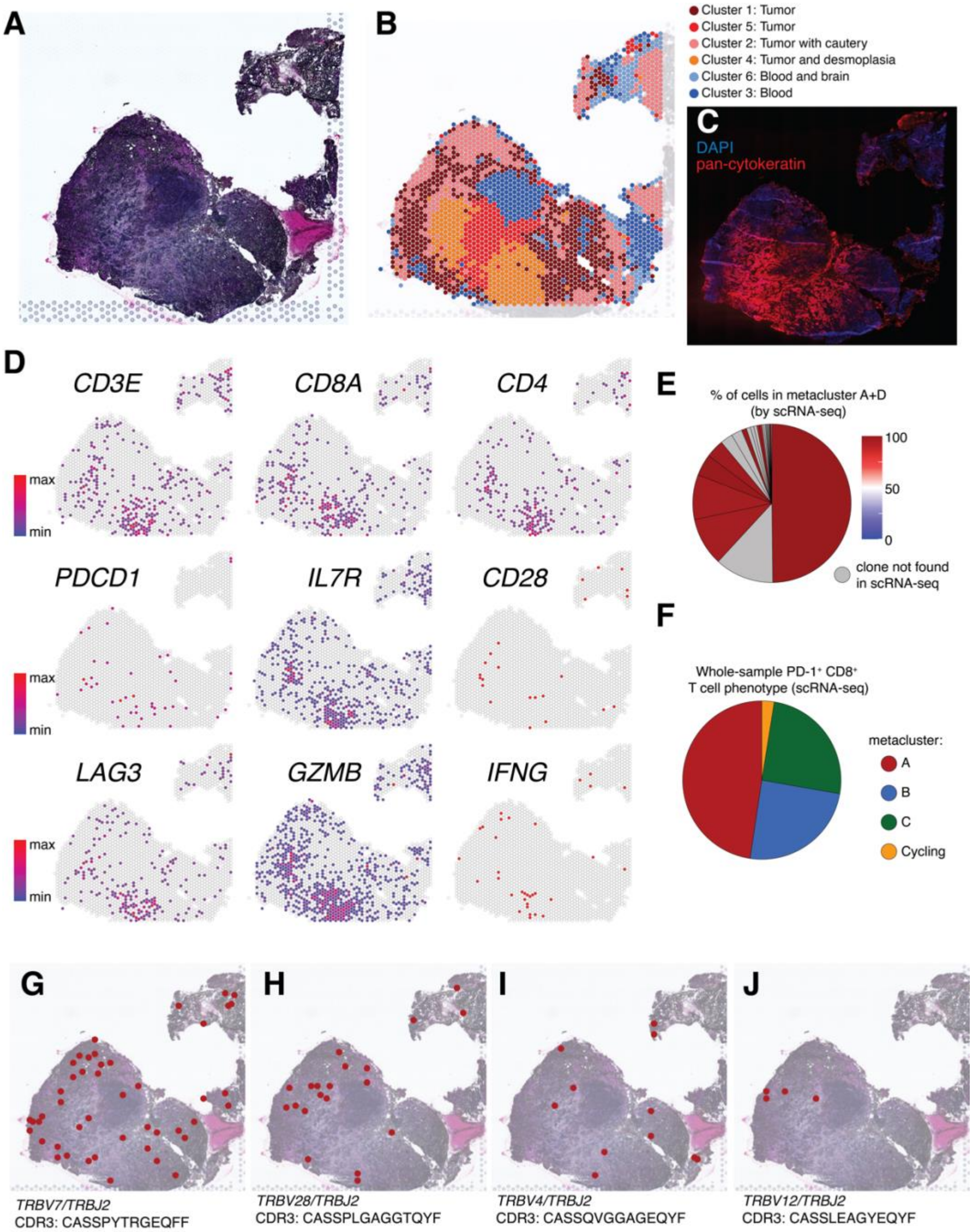

Figure S10: Spatial gene expression and TCR sequencing of a lung adenocarcinoma brain metastasis (patient 15), related to Figures 6-7. (A) H&E staining of the metastasis. (B) Gene expression clusters identified by graph-based clustering. (C) Immunofluoresence of an adjacent section with DAPI and pan-cytokeratin (a tumor marker) demonstrates this tissue section is comprised entirely of tumor parenchyma. (D) Spatial expression of selected immune-related genes. (E) Spatial TCR sequencing was performed on this tumor section. Shown are the T cell clones identified within this section, colored by their scRNA-seq phenotype, if applicable. Size the of pie chart slice indicates TCR frequency among spatially-identified clones. All clones found both in scRNA-seq and spatial TCR sequencing were composed of terminally-differentiated cells from metaclusters A and D. (F) scRNA-seq metacluster of all cells sequenced from the matched patient sample. When compared to panel (E), terminally differentiated cells were highly enriched in the tumor parenchyma. (G-J) Spatial distribution of four example TCR clones throughout the tissue section.

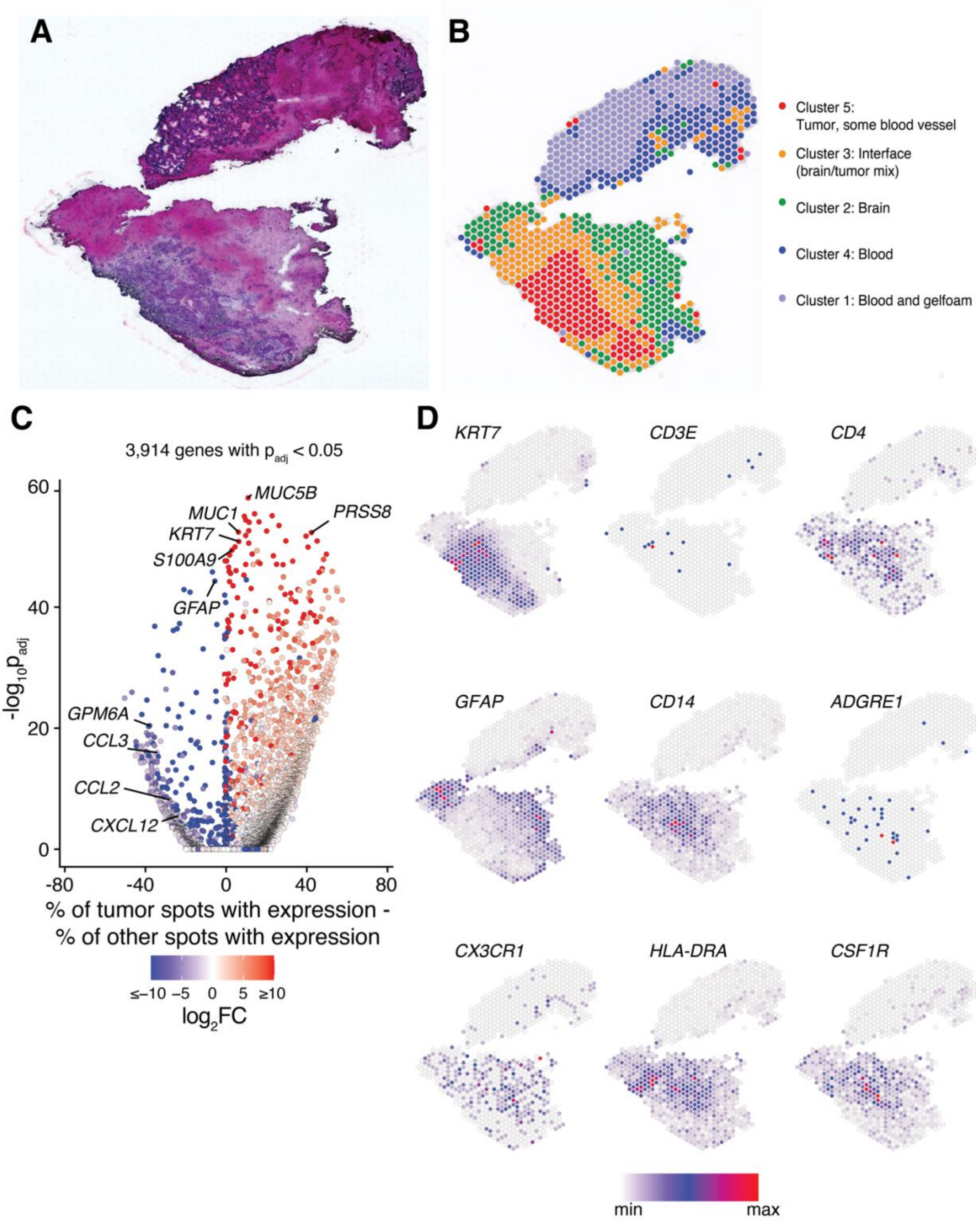

Figure S11: Spatial gene expression of a lung adenocarcinoma brain metastasis tissue section poorly infiltrated by CD8⁺ T cells (patient 19), related to Figure 6. (A) H&E staining of a lung adenocarcinoma brain metastasis. (B) Spatial distribution of annotated gene expression clusters. (C) Differences in gene expression between capture spots with tumor (cluster 5) and all other clusters. (D) Spatial expression of selected genes shows high expression of *KRT7*, a cytokeratin protein, within the tumor. T cell infiltrate (*CD3E*) is sparse and located at the brain/tumor interface, but high levels of innate immune-associated genes such as *CD14* are also found around the tumor. *GFAP*, expressed in glial cells such as astrocytes, is expressed beyond the tumor/brain interface.

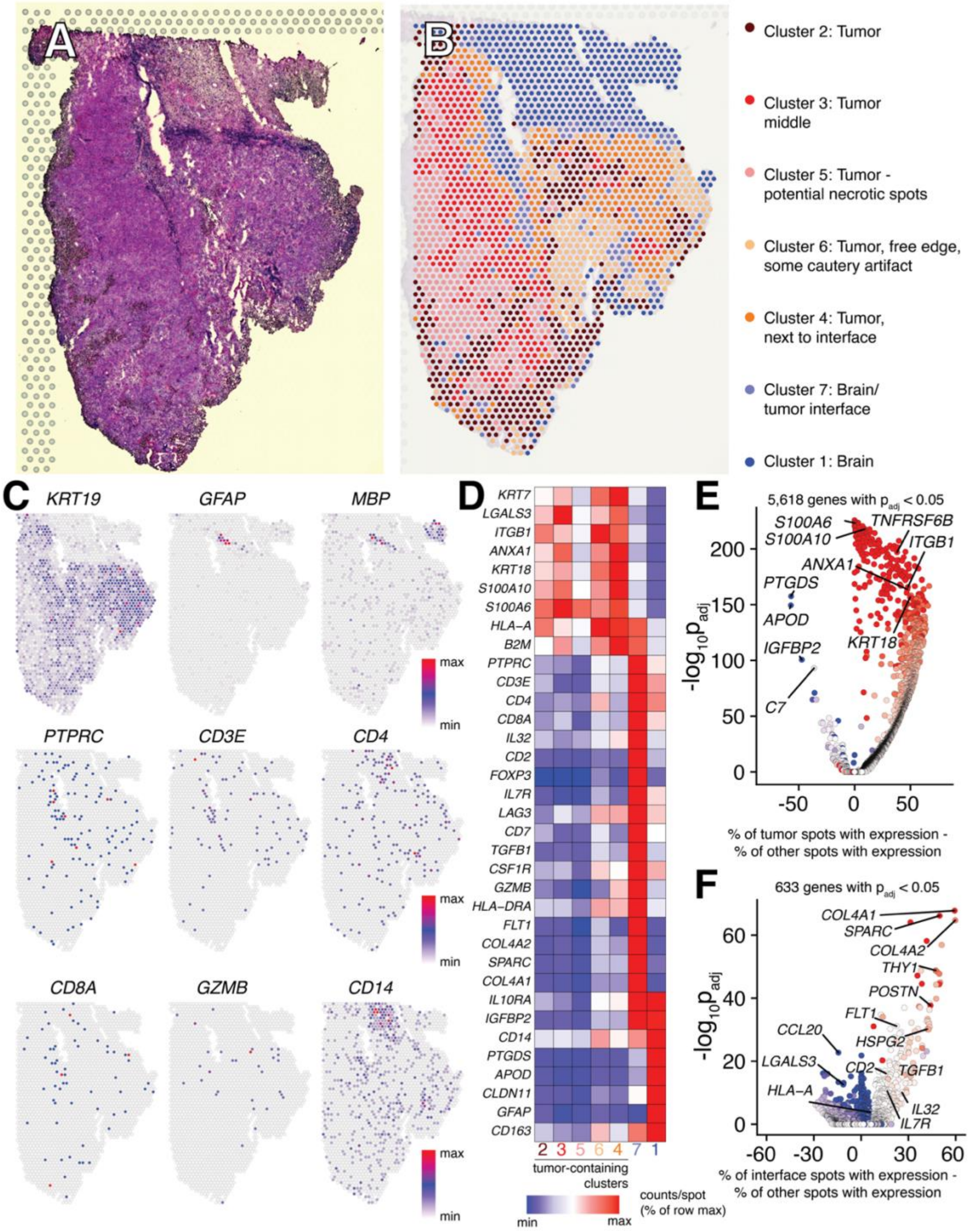

Figure S12: Spatial gene expression within a lung adenocarcinoma tissue section (patient 26), related to Figure 6. (A) H&E staining of a lung adenocarcinoma brain metastasis. (B) Spatial distribution of annotated gene expression clusters. (C) Expression of selected genes, including *KRT19* (a cytokeratin and tumor marker), *GFAP*, and *MBP* (myelin basic protein, expressed by Schwann cells and oligodendrocytes). (D) Heatmap of selected gene expression density by cluster. (E) Volcano plot of gene expression differences in tumor spots (clusters 2, 3, 4, 5, and 6) compared to other tissue spots. (F) Gene expression differences at the brain/tumor interface (cluster 7) compared to all other spots.

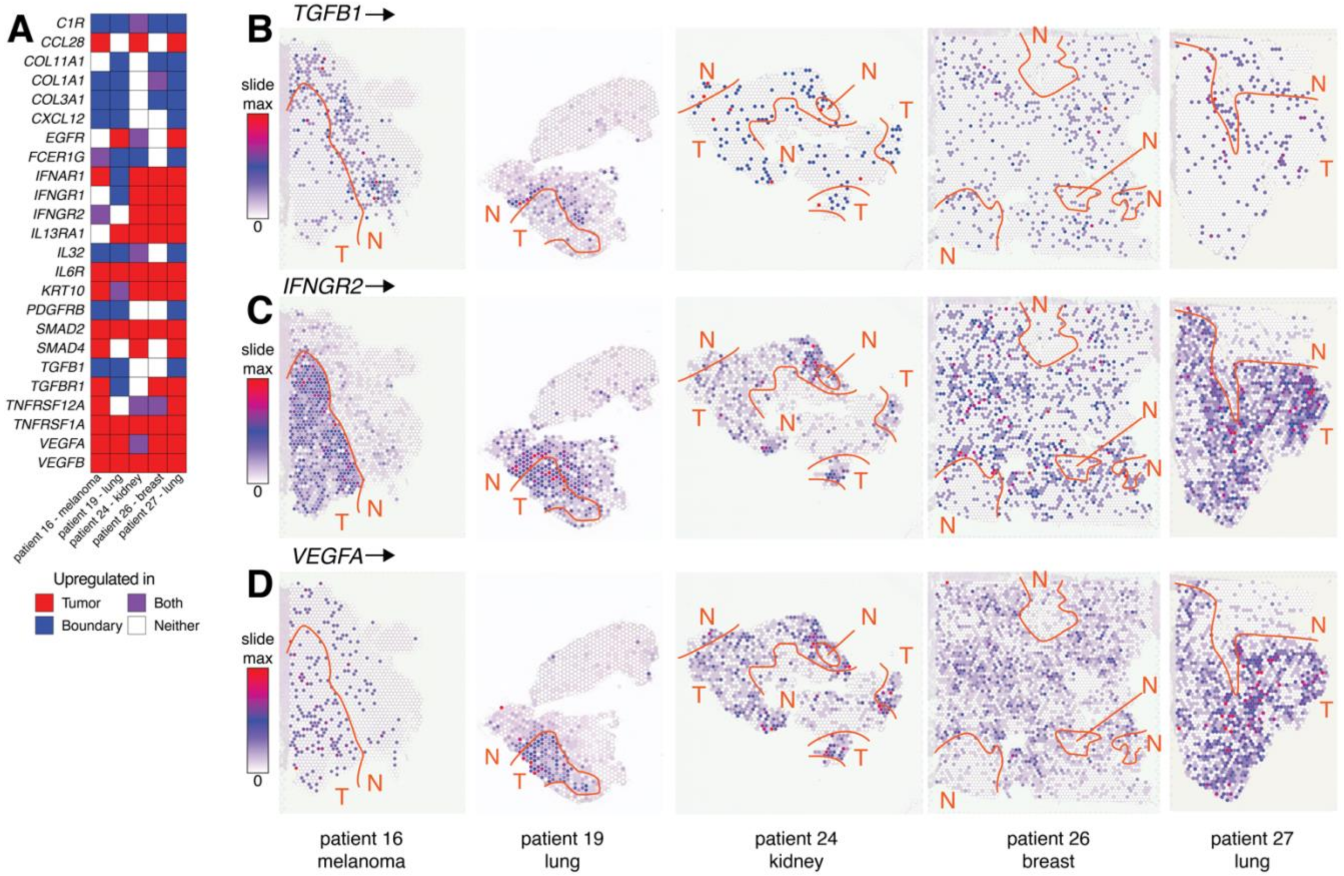

Figure S13: Spatial location of cytokine, chemokine, and their receptor genes in human brain metastases, related to Figure 6. (A) Heatmap showing sites of selected gene upregulation in five brain metastases samples. Sample 15 was excluded from analysis as it contained only tumor parenchyma with no margin (Fig. S10C). Upregulation is defined as higher expression and an adjusted p-value of <0.05 in the tumor, boundary, or both compared to all other tissue spots. (B-D) Spatial expression of *TGFB1* (encoding TGF-β), *IFNGR2* (encoding the β chain of the IFN-γ receptor), and *VEGFA*, which encodes the Vascular Endothelial Growth Factor A. Tissue annotations for approximate regions of tumor (T) or non-tumor (N) are shown. See Figs. 6, S10, S11, S13, and S14 for more detailed tissue annotation.

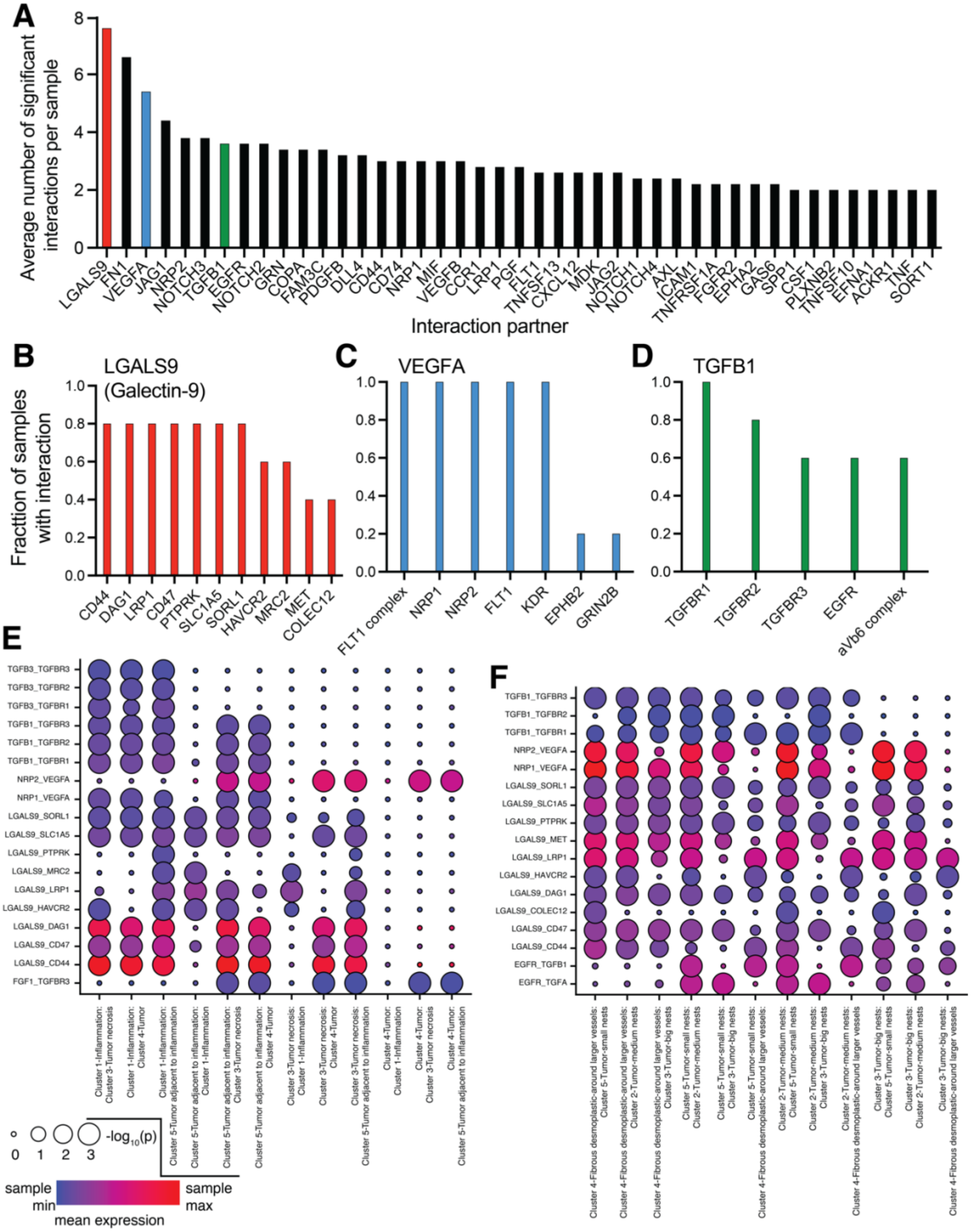

Figure S14: CellPhoneDB analysis reveals signaling pathways present in the brain metastasis microenvironment, related to Figure 6. CellPhoneDB was performed on the five spatial transcriptomics samples on which normal and tumor tissue was present (patients 16, 19, 24, 26, and 27), using pathologist-annotated gene expression clusters to group capture regions. (A) Interaction partners with the highest number of annotated and statistically-significant interactions between gene expression clusters. (B-D) Interaction partners of LGALS9 (Galectin-9), VEGFA, and TGFB1 (TGF-β). (E-F) Interactions of Galectin-9, VEGFA, and TGF-β among selected gene expression clusters in patient 16 and 24, respectively. To plot the logarithm of low p-values, 0.001 was added to each p-value calculated by CellPhoneDB.
